# Correction: A Continuous-Exchange Cell-Free Protein Synthesis System Based on Extracts from Cultured Insect Cells

**DOI:** 10.1371/journal.pone.0100344

**Published:** 2014-06-17

**Authors:** The PLOS ONE Staff

## Notice of Republication

This article was republished on May 21, 2014, to correct a typesetting error in the author byline and citation. The publisher apologizes for the error. Please download this article again to view the correct version. The originally published, uncorrected article and the republished, corrected article are provided here for reference.

## Supporting Information

File S1 Originally published, uncorrected article.
(PDF)

File S2 Republished, corrected article.
(PDF)
