# Supplementary figures and images for: A Continuous-Exchange Cell-Free Protein Synthesis System Based on Extracts from Cultured Insect Cells
Source: PLoS One. 2014 May 7;9(5):e96635. doi: 10.1371/journal.pone.0096635 (PMC4013096; doi:10.1371/journal.pone.0096635)

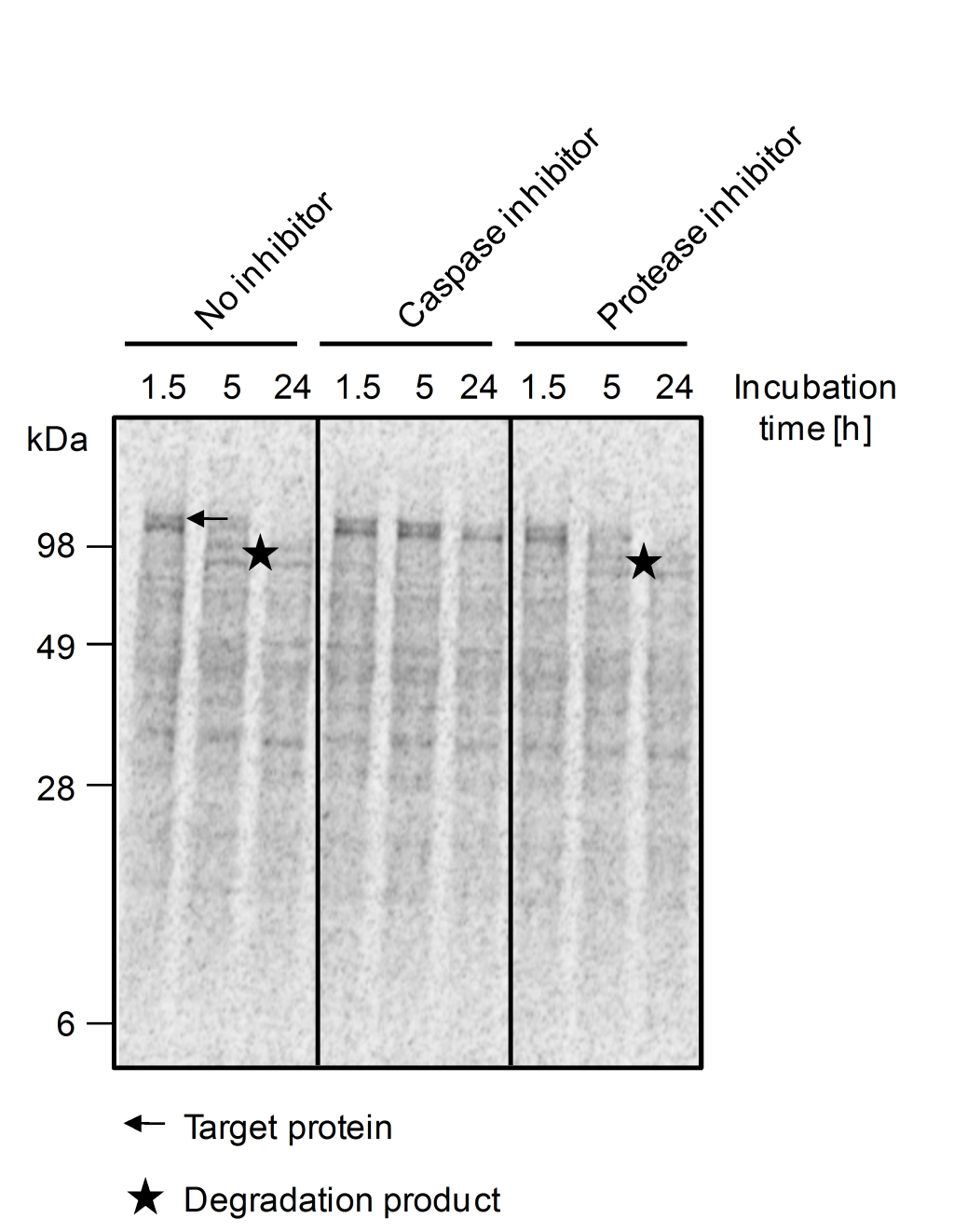

Supplement: Figure S1 — Influence of caspase inhibitor on the stability of de novo synthesized membrane proteins. Cell-free synthesis of the epidermal growth factor receptor (fused to a melittin signal peptide at the N-Terminus and eYFP at the C-Terminus, 163 kDa) was performed using the coupled insect cell-free system in batch mode in presence of 14C-leucine and in presence and absence of caspase inhibitor Z-VAD-FMK. Reactions were stopped at the indicated incubation times. In addition, one reaction was performed in presence of a commercially available protease inhibitor mix (“Complete protease inhibitor cocktail EDTA-free”, Roche). Synthesized proteins were subsequently analyzed by SDS-PAGE and autoradiography. (TIF) [file pone.0096635.s001.tif]

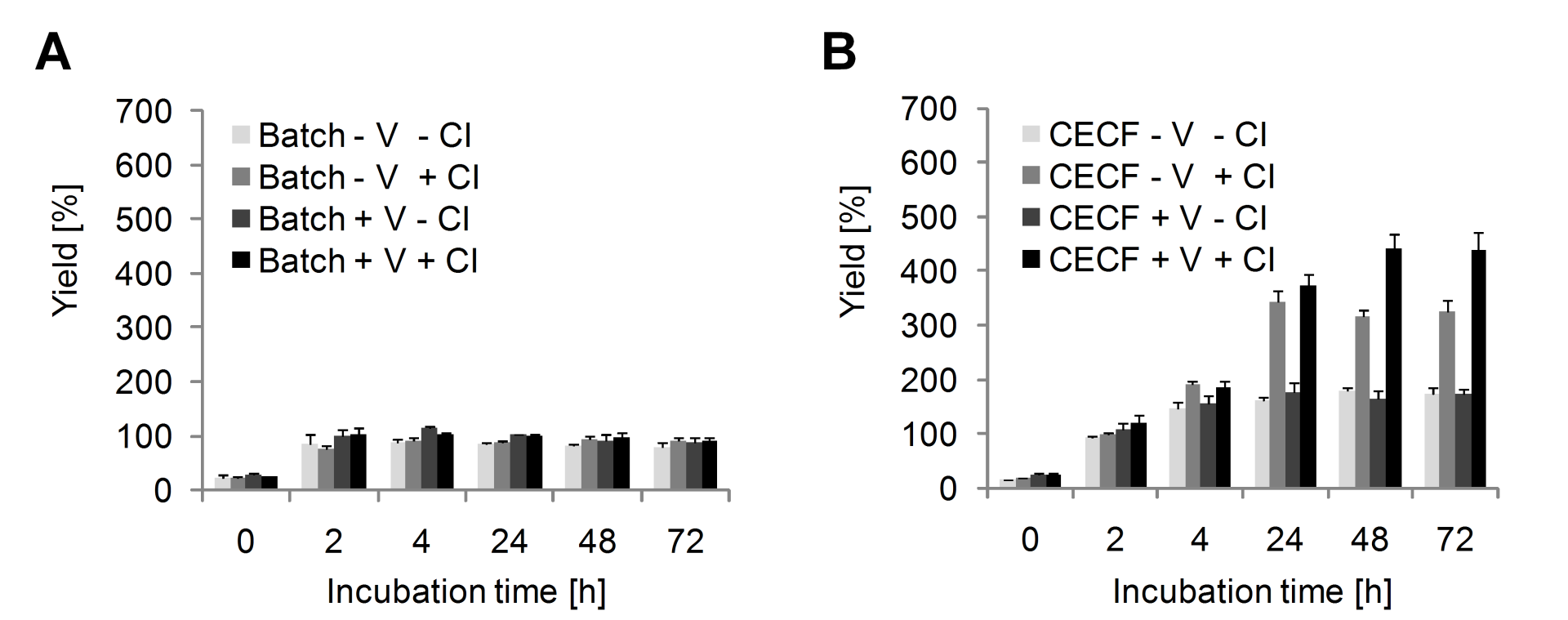

Supplement: Figure S2 — Time course of batch-based (A) and CECF-based (B) expression of eYFP. eYFP was synthesized using an in vitro translation system based on insect lysates in the presence of 14C-leucine. Quantification of de novo synthesized eYFP was performed by liquid scintillation counting. Protein yields of eYFP are shown in percent with the concentration of the target protein determined after 2 h of incubation set as 100% (batch, + V, - CI). Standard deviations were calculated from triplicate analysis (n = 3). (TIF) [file pone.0096635.s002.tif]

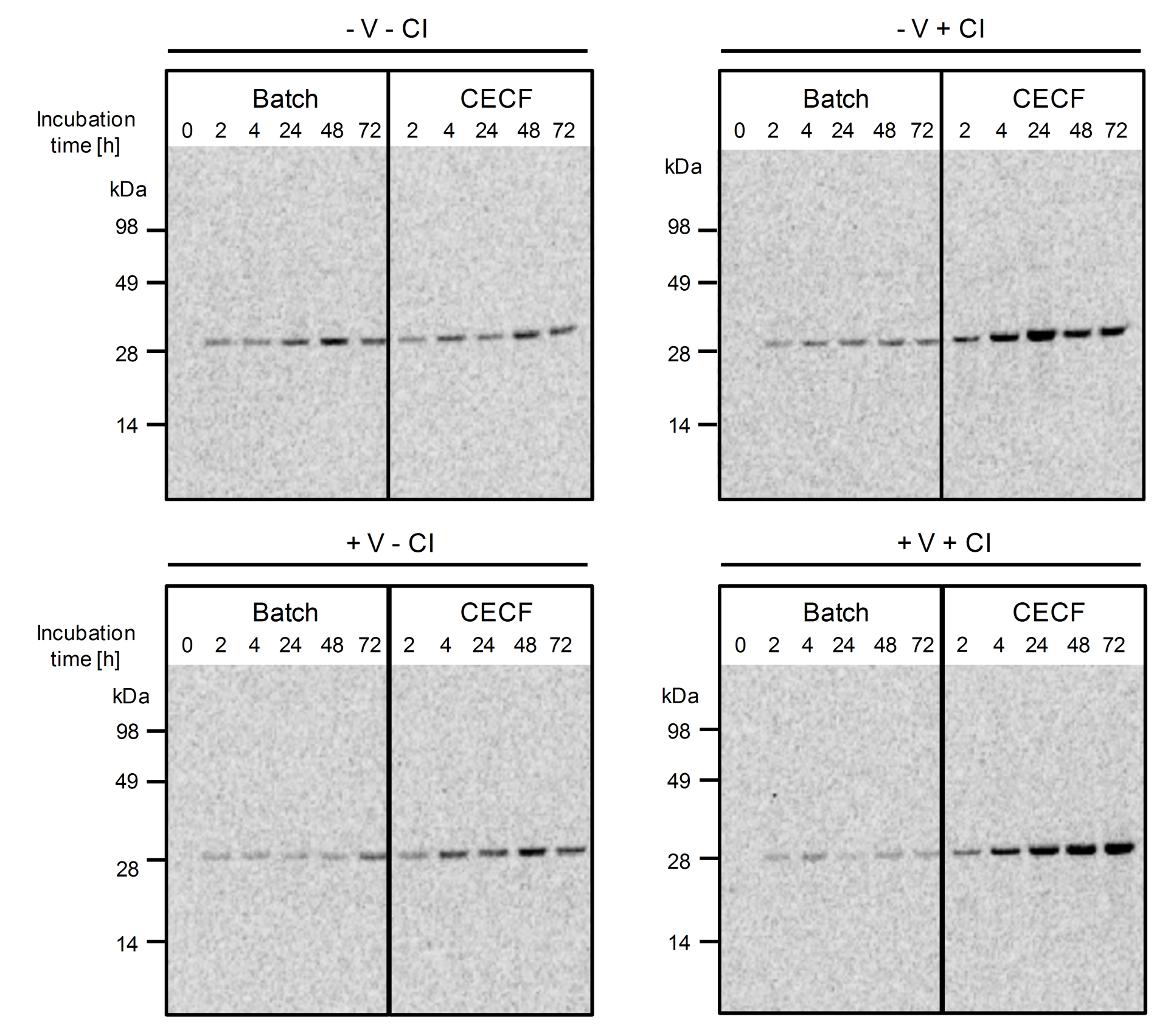

Supplement: Figure S3 — Time course of 14C-leucine labeled eYFP (supernatant fraction) synthesized in batch and CECF mode. Cell-free reactions using insect lysate were carried out in the presence (+) and absence (−) of insect vesicles (V) and caspase inhibitor (CI). Synthesized proteins were analyzed by SDS-PAGE and autoradiography. Cell-free synthesized eYFP shows a migration pattern corresponding to its expected molecular mass (calculated molecular mass = 29 kDa). (TIF) [file pone.0096635.s003.tif]

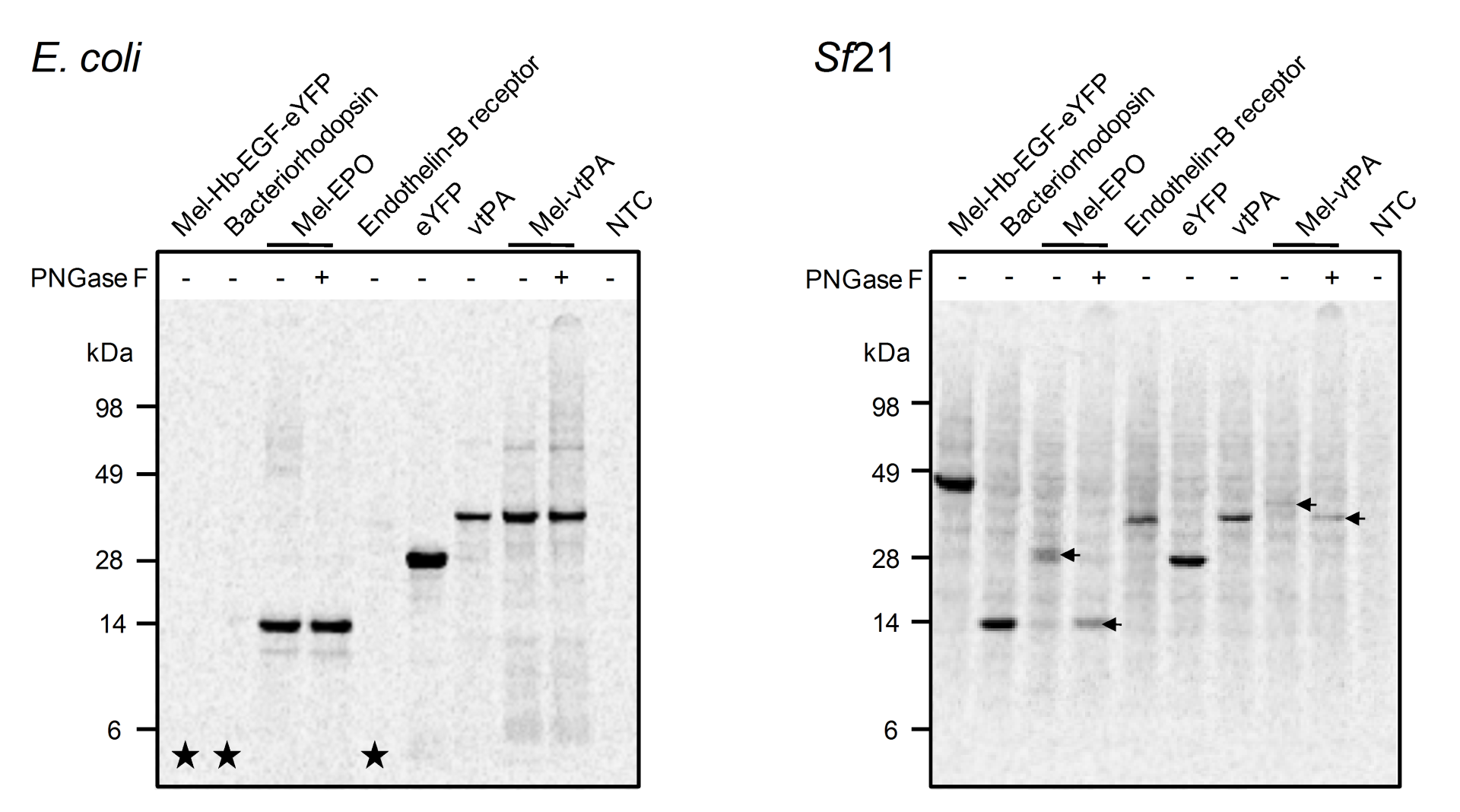

Supplement: Figure S4 — Comparative expression of representative model proteins in E. coli and insect cell-free systems. Cell-free synthesis of model proteins was performed using the batch-based E. coli cell-free system (EasyXpress Protein Synthesis Kit, Qiagen) and the vesicle-containing insect cell-free system in presence of 14C-leucine. Synthesized proteins were analyzed by SDS-PAGE and autoradiography. Aliquots of the glycoproteins Mel-EPO and Mel-vtPA were subjected to digestion with (+) PNGase F. Black arrows indicate the shift in protein size between glycosylated proteins and proteins after degylcosylation with PNGase F. Only in case of Mel-EPO and Mel-vtPA synthesized in the insect cell-free system, digestion with PNGase F led to a visible reduction of the protein's molecular mass, indicating the successful glycosylation of these proteins. Asterisks are marking the proteins were expression in E. coli lysate failed. NTC = No template control; translation reaction without addition of a DNA template. (TIF) [file pone.0096635.s004.tif]

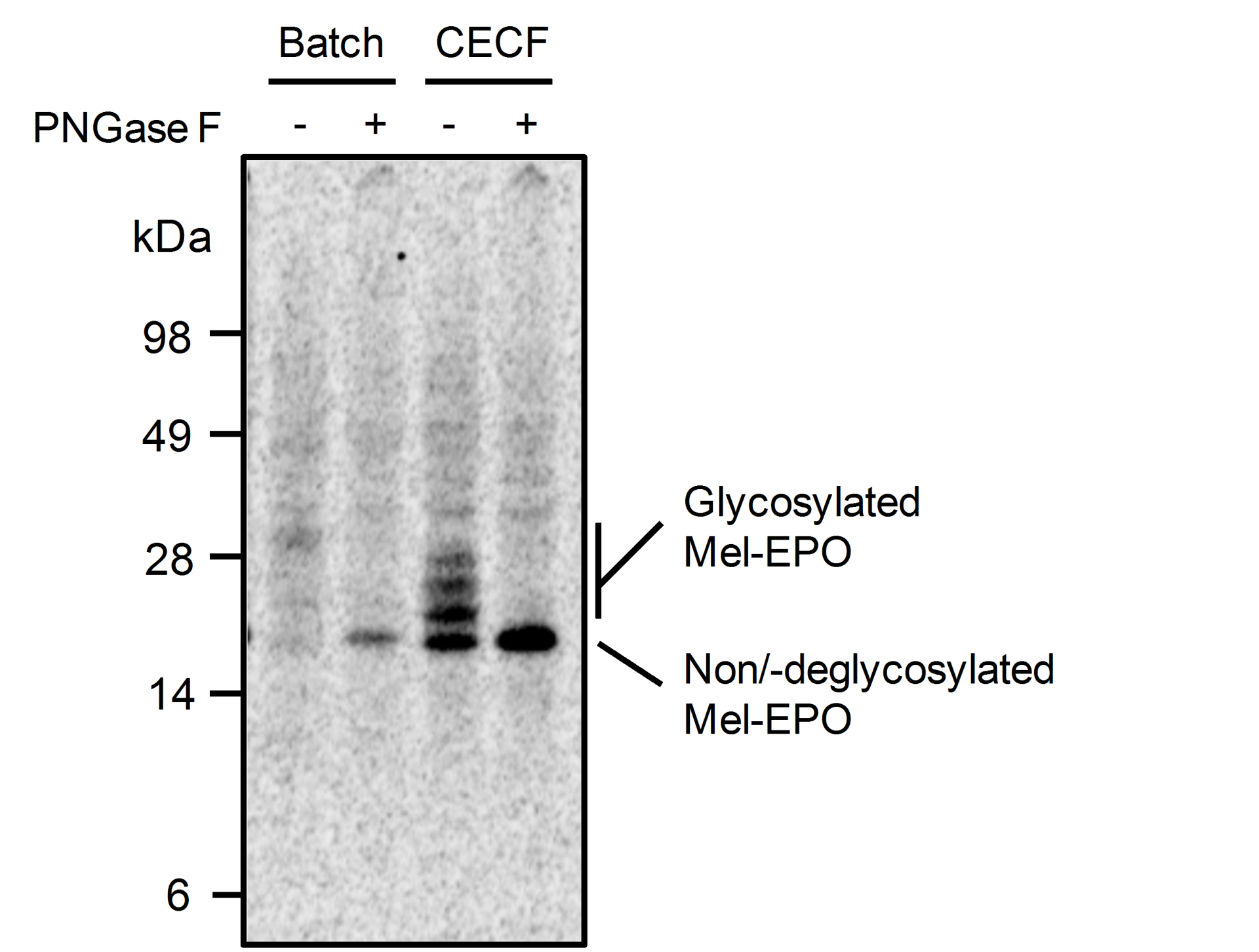

Supplement: Figure S5 — Glycosylation analysis of cell-free expressed Mel-EPO. Cell-free reactions were carried out in the presence of caspase inhibitor, 14C-leucine and DTT using the insect batch and CECF system (48 h). The autoradiograph shows Mel-EPO in its glycosylated and non-glycosylated form. In addition, a deglycosylation assay was performed using PNGase F. (TIF) [file pone.0096635.s005.tif]

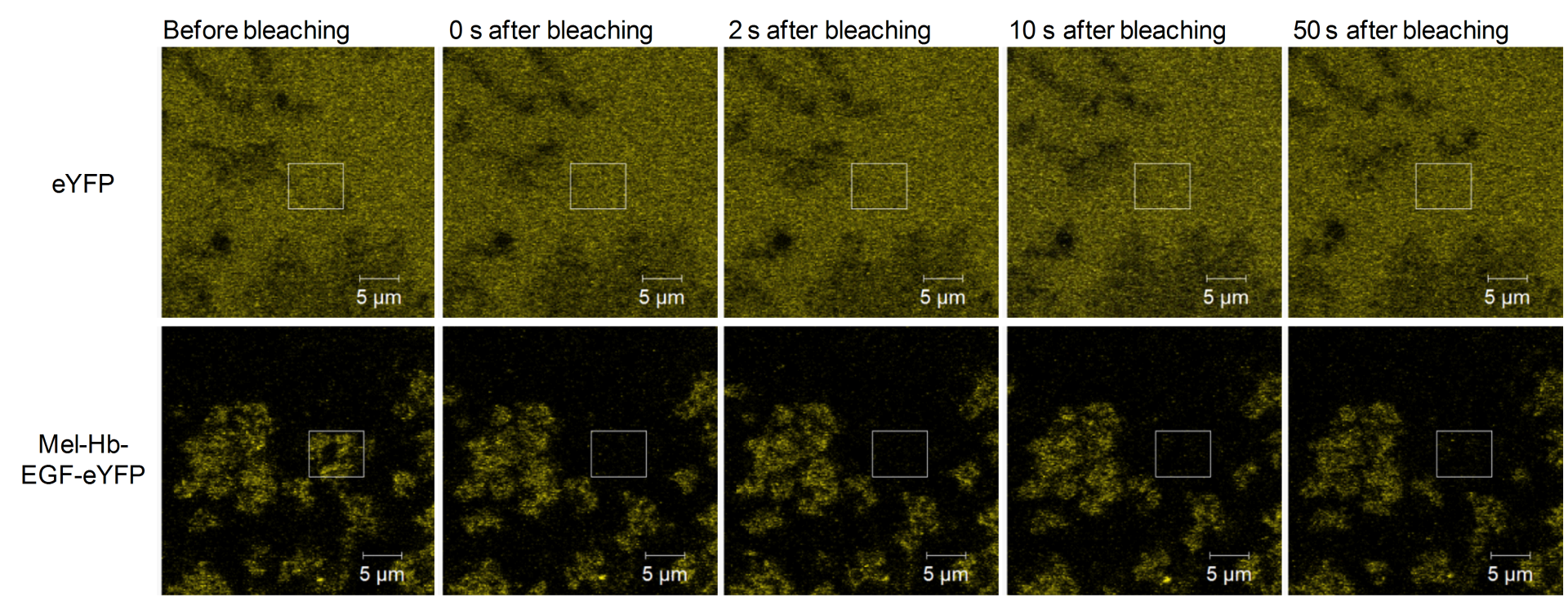

Supplement: Figure S6 — FRAP analysis of the type-I transmembrane protein Mel-Hb-EGF-eYFP and the fluorescent and soluble protein eYFP. Both proteins were synthesized in standard batch reactions. For confocal microscopy, translation mixtures were excited at 488 nm while fluorescence emission was recorded with a longpass filter in the wavelength range above 505 nm (LSM 510 Meta, Zeiss). Strong emission intensity of de novo synthesized Mel-Hb-EGF-eYFP was observed in microsomal structures. In contrast to this observation, fluorescence of eYFP was observed in the cytosolic fraction of the lysate surrounding the vesicles. Samples were analyzed before and after photobleaching. Fluorescent vesicles of Mel-Hb-EGF-eYFP were bleached completely after 500 iterations with 100% laser intensity (argon laser, 488 nm). As expected, no fluorescence recovery was observed after 50 s of incubation, indicating that membrane-embedded Mel-Hb-EGF-eYFP was not delivered by diffusion from the cytosolic surrounding. In contrast, laser exposure of the cytosolic protein eYFP did not result in a detectable photobleaching effect. (TIF) [file pone.0096635.s006.tif]

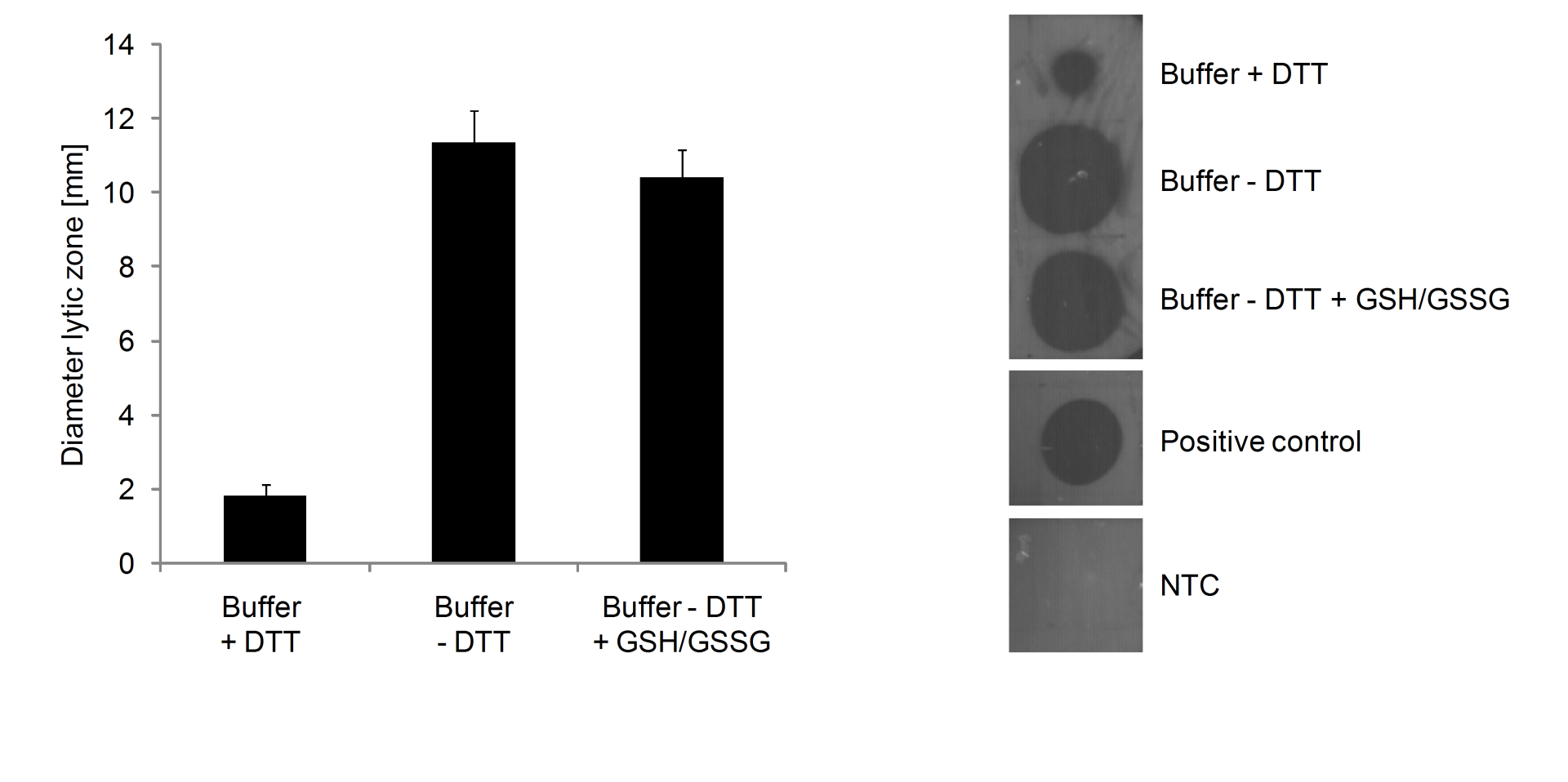

Supplement: Figure S7 — Analysis of Mel-vtPA expression and activity. Synthesis of Mel-vtPA was performed in batch mode for 2 h using three different reaction buffers [(1) buffer with DTT; (2) buffer without DTT; (3) buffer without DTT, but supplemented with GSH and GSSG] in combination with insect lysate without DTT, but supplemented with GSH and GSSG. Activity of Mel-vtPA was analyzed using the fibrin-agarose-plate assay. All samples were diluted to 0.5 µg/ml. NTC = No template control; translation reaction without addition of a DNA template. Positive control = Purified full length tPA (Anaspec) (0.5 µg/ml). Standard deviations were calculated from triplicate analysis (n = 3). (TIF) [file pone.0096635.s007.tif]
