# Supplementary material for: A Continuous-Exchange Cell-Free Protein Synthesis System Based on Extracts from Cultured Insect Cells
Source: PLoS One. 2014 May 7;9(5):e96635. doi: 10.1371/journal.pone.0096635 (PMC4013096; doi:10.1371/journal.pone.0096635)
Supplement: Table S1 — List of cell-free expressed model proteins. (DOCX) [file pone.0096635.s008.docx]

Table S1. List of cell-free expressed model proteins.

| **Abbreviation** | **Protein name** | **Modification** | **Classification** | **Molecular mass** |
| --- | --- | --- | --- | --- |
| eYFP | Enhanced yellow fluorescent protein (*Aequorea victoria*) | N-terminally fused to a Strep-tagII sequence | Fluorescent soluble protein, cytosolic | 29 kDa |
| Mel-Hb-EGF-eYFP | Proheparin-binding EGF-like growth factor *(Rattus norvegicus)* | N-terminally fused to a melittin signal sequence and C-terminally fused to eYFP | Type-I transmembrane protein | 51 kDa |
| - | Bacteriorhodopsin *(Halobacterium salinarum)* | Codon-optimized for expression in *Sf*21 cells | Transmembrane protein with seven membrane spanning helices | 27 kDa |
| Mel-EPO | Erythropoietin *(Homo sapiens)* | N-terminally fused to a melittin signal sequence | Glycoprotein with three N-glycosylation sites and one O-glycosylation site | 21 kDa |
| - | Endothelin-B receptor *(Homo sapiens)* | - | G-protein coupled receptor, seven transmembrane domains | 49 kDa |
| Mel-vtPA | Truncated tissue-type-plasminogen activator *(Homo sapiens)* | N-terminally fused to a melittin signal sequence | Glycoprotein with nine disulfide bonds | 41 kDa |
