# Supplementary material for: Correction: A Continuous-Exchange Cell-Free Protein Synthesis System Based on Extracts from Cultured Insect Cells
Source: PLoS One. 2014 Jun 17;9(6):e100344. doi: 10.1371/journal.pone.0100344 (PMC4061124; doi:10.1371/journal.pone.0100344)
Supplement: File S2 — Republished, corrected article. (PDF) [file pone.0100344.s002.pdf]

OPEN ACCESS Freely available online

PLOS | ONE

A Continuous-Exchange Cell-Free Protein Synthesis System Based on Extracts from Cultured Insect Cells

Marlitt Stech[1], Robert B. Quast[1], Rita Sachse[1], Corina Schulze[2], Doreen A. Wüstenhagen[1], Stefan Kubick[1]*

1 Fraunhofer Institute for Biomedical Engineering (IBMT), Branch Potsdam-Golm, Potsdam, Germany, **2** Beuth Hochschule für Technik Berlin - University of Applied Sciences Berlin, Life Sciences and Technology, Berlin, Germany

Abstract

In this study, we present a novel technique for the synthesis of complex prokaryotic and eukaryotic proteins by using a continuous-exchange cell-free (CECF) protein synthesis system based on extracts from cultured insect cells. Our approach consists of two basic elements: First, protein synthesis is performed in insect cell lysates which harbor endogenous microsomal vesicles, enabling a translocation of *de novo* synthesized target proteins into the lumen of the insect vesicles or, in the case of membrane proteins, their embedding into a natural membrane scaffold. Second, cell-free reactions are performed in a two chamber dialysis device for 48 h. The combination of the eukaryotic cell-free translation system based on insect cell extracts and the CECF translation system results in significantly prolonged reaction life times and increased protein yields compared to conventional batch reactions. In this context, we demonstrate the synthesis of various representative model proteins, among them cytosolic proteins, pharmacological relevant membrane proteins and glycosylated proteins in an endotoxin-free environment. Furthermore, the cell-free system used in this study is well-suited for the synthesis of biologically active tissue-type-plasminogen activator, a complex eukaryotic protein harboring multiple disulfide bonds.

Citation: Stech M, Quast RB, Sachse R, Schulze C, Wüstenhagen DA (2014) A Continuous-Exchange Cell-Free Protein Synthesis System Based on Extracts from Cultured Insect Cells. PLoS ONE 9(5): e96635. doi:10.1371/journal.pone.0096635

Editor: Thomas Preiss, The John Curtin School of Medical Research, Australia

Received January 20, 2014; **Accepted** April 9, 2014; **Published** May 7, 2014

Copyright: © 2014 Stech et al. This is an open-access article distributed under the terms of the Creative Commons Attribution License, which permits unrestricted use, distribution, and reproduction in any medium, provided the original author and source are credited.

Funding: This research is supported by the German Ministry of Education and Research (BMBF, No. 0315942 and No. 0312039). The funders had no role in study design, data collection and analysis, decision to publish, or preparation of the manuscript.

Competing Interests: The authors have declared that no competing interests exist.

* E-mail: stefan.kubick@ibmt.fraunhofer.de

Introduction

Over the last decade, cell-free methods have proven themselves as a valuable platform allowing the synthesis of many different protein classes including membrane proteins [1,2,3,4,5,6,7], proteins with posttranslational modifications [8,9,10,11,12,13,14] and even toxic proteins [15,16,17]. Many problematic issues connected with a cell-based expression of proteins, such as protein insolubility and toxicity, can be circumvented by the use of tailor-made cell-free expression systems.

In addition to prokaryotic systems, eukaryotic cell-free systems have proven to accelerate the production of functional proteins [18,19]. Wheat germ extracts, for example, are highly productive and reach protein yields comparable to *Escherichia coli (E. coli)* - based systems [20,21]. But still, expression in *E. coli* and wheat germ systems has their limitations when it comes to the synthesis of complex proteins and proteins which require co-translational and posttranslational modifications [9,22]. Covalent posttranslational modifications such as glycosylation and disulfide bond formation are very common among eukaryotic proteins and it is well-known that they have a great impact on protein folding, localization and activity [23]. One of the main demands and also challenges of cell-free systems is to produce functional proteins. Thus, it is of highest interest to develop cell-free translation systems that ensure the formation of posttranslational modifications while providing a sufficient amount of protein for further functional and structural analysis.

The cell-free system used in this study is based on translationally active lysates from cultured *Spodoptera frugiperda* (*Sf*21) cells [13,24,25]. Due to a very gentle lysate preparation procedure, structures from the endoplasmic reticulum (ER) can be maintained in the lysate as vital membranous vesicles. These vesicles are translocationally active and provide a suitable scaffold for membrane protein embedding into a natural lipid bilayer [26,27]. Furthermore, due to their presence in the lysate, different types of posttranslational modifications can be performed on proteins, such as glycosylation, signal peptide cleavage [13], lipidation [14], phosphorylation [28] and disulfide bond formation [29,30,31].

Until today, cell-free reactions using the vesicle-containing insect expression system have been performed in batch format. On the one hand, batch-based reactions are easy-to-handle and enable a fast and reliable synthesis of a given target protein. On the other hand, the rapid depletion of energy resourses and the accumulation of inhibitory by-products such as free phosphates usually lead to a short life time of the system resulting in limited protein yields [32]. The introduction of the continuous-flow cell-free (CFCF) system [33,34] and later, the continuous-exchange cell-free (CECF) system [35] helped to overcome these problems. Both expression formats exhibit an extended reaction life time, but due to the operational complexity of the CFCF system, CECF systems were more favorably applied [36]. The basis of the latter system is a passive exchange of energy resources and by-products through a semi-permeable dialysis membrane that separates the reaction

compartment, the place of protein synthesis, from the feeding compartment, leading to prolonged reaction life times and significantly increased protein yields [35,37]. In this publication, we introduce the combination of our coupled cell-free translation system based on insect lysates and a commercially available CECF device. We demonstrate that representative model proteins, including membrane proteins, proteins with posttranslational modifications and cytosolic proteins, can be produced in the novel eukaryotic CECF system with significantly increased protein yields compared to batch-based reactions. Moreover, using the insect CECF system, we were able to produce biologically active human tissue-type-plasminogen activator, a complex eukaryotic protein harboring multiple intramolecularly formed disulfide bonds.

Materials and Methods

Template generation

The following proteins (Table S1) were selected as model proteins to evaluate the performance of the insect CECF system: (1) eYFP (29 kDa), a soluble fluorescent protein harboring a Strep-tagII sequence at the N-terminus, (2) proheparin-binding EGF-like growth factor, a type-I transmembrane protein fused to a melittin signal peptide (Mel) at the N-Terminus and eYFP at the C-Terminus (Mel-Hb-EGF-eYFP, 51 kDa); (3) bacteriorhodopsin (27 kDa), a prokaryotic transmembrane protein featuring seven membrane spanning helices, (4) human erythropoietin (EPO, 21 kDa, protein in its non-glycosylated form), a glycoprotein that bears three N-glycosylation sites and one O-glycosylation site, (5) endothelin-B receptor (49 kDa), a human G-protein coupled receptor and (6) a recombinant variant of tissue-type-plasminogen activator (vtPA, 41 kDa). vtPA is a truncated variant of full length tPA which consists of only two domains, the kringle-2 domain and the catalytic domain but lacks the N-terminal finger domain, the epidermal growth factor-like domain and the kringle-1 domain [38,39,40]. vtPA contains nine disulfide bonds and two potential sites for the addition of N-linked oligosaccharides. Genes of EPO and vtPA were fused to a melittin signal sequence (Mel-EPO, Mel-vtPA) in order to favor an efficient translocation of cell-free synthesized proteins into the endoplasmic reticulum-derived vesicles of the insect lysate. Batch and CECF reactions were performed using plasmids as DNA templates. Plasmids were generated from PCR-amplified genes which were cloned into appropriate expression vectors (pIX3.0, Qiagen; pIX2.0, RiNA GmbH). Constructs used for cell-free protein synthesis contained a T7 promoter sequence upstream of the gene's open reading frame and a T7 terminator sequence downstream of the deduced stop codon. The following constructs were used in this study: pIX3.0-eYFP, pIX3.0-Mel-Hb-EGF-eYFP, pMA-bacteriorhodopsin (GeneArt Gene Synthesis, Life technologies), pIX2.0-Mel-EPO, pIX3.0-endothelin-B receptor, pIX3.0-vtPA and pIX3.0-Mel-vtPA.

Cell-free protein synthesis

Prokaryotic cell-free protein synthesis. Cell-free expression in *E. coli* cell lysates was performed using the EasyXpress Protein synthesis Kit (Qiagen) following the manufacturer's instructions. For qualitative and quantitative analysis of *de novo* synthesized proteins, reactions were supplemented with ^{14}C-labeled leucine (25 μM) (PerkinElmer) yielding a specific radioactivity of 2 dpm/pmol.

Eukaryotic cell-free protein synthesis. Insect lysate preparation procedure. Translationally active lysates from cultured *Sf*21 cells were generated as described previously [13]. The depicted procedure was modified by using a homogenization buffer without the reducing agent DTT, but supplemented with GSH and GSSG (0.5 mM each). Insect lysate without vesicles was prepared in the following way: Aggregates of endoplasmic reticulum-derived insect vesicles were separated from the cytosolic fraction of the lysate by centrifugation at 16,000×g for 10 min at 4°C. After centrifugation, the lysate supernatant was separated immediately from the vesicular fraction. Insect lysate without vesicles was used directly or frozen in liquid nitrogen and stored at −80°C.

Coupled transcription-translation procedure using insect lysate. Coupled transcription-translation reactions were performed using 40% insect lysate supplemented with HEPES-KOH (final concentration, f.c. 30 mM, pH 7.6; Merck), $Mg(OAc)_2$ (f.c. 2.5 mM; Merck), KOAc (f.c. 75 mM; Merck), amino acids (complete 100 μM f.c.; Merck), spermidine (f.c. 0.25 mM; Roche) and energy regenerating components (f.c. 1.75 mM ATP, 0.3 mM GTP; Roche). In order to allow transcription of mRNA CTP (f.c. 0.3 mM; Roche), UTP (f.c. 0.3 mM; Roche), T7 RNA polymerase (f.c. 1 U/μl; Agilent) and the cap analogue G(ppp)G (f.c. 0.33 mM; Prof. Edward Darzynkiewicz, Warsaw University) were added to the protein synthesis reaction. Initial tests using different cap analogues, among them being m^7G(ppp)G and G(ppp)G, demonstrated that both, methylated and unmethylated cap analogues can be successfully applied in the coupled transcription-translation reaction. Since the unmethylted cap analogue G(ppp)G is cheaper compared to the methylated one, G(ppp)G was used for further studies. To monitor protein quality and quantity, translation mixtures were supplied with ^{14}C-labeled leucine (f.c. 11–35 μM, depending on the individual experiment) (PerkinElmer) yielding a specific radioactivity of 10–45 dpm/pmol. Translation was initiated by the addition of plasmid in a final concentration of 60 μg/ml. Batch reactions were performed in a 50 μl reaction volume in a thermomixer (Thermomixer comfort, Eppendorf) at 27°C for up to 48 h with gentle agitation at 600 rpm.

For CECF reactions a commercially available two-chamber dialysis device (5PRIME) was used that consists of a 50 μl reaction and a 1000 μl feeding chamber which are separated by a semi-permeable dialysis membrane (cut-off 10 kDa). Two mixes, the reaction and the feeding mixture, were prepared separately from each other and filled one after another into the device. The reaction mixture (total volume 50 μl) was prepared similarly as a standard batch-based reaction (see above). The feeding mixture (total volume 1000 μl) was composed of HEPES-KOH (f.c. 30 mM, pH 7.6; Merck), $Mg(OAc)_2$ (f.c. 2.5 mM; Merck), KOAc (f.c. 75 mM; Merck), amino acids (complete 100 μM f.c.; Merck), spermidine (f.c. 0.25 mM; Roche), energy regenerating components (f.c. 1.75 mM ATP, 0.3 mM GTP; Roche), CTP (f.c. 0.3 mM; Roche), UTP (f.c. 0.3 mM; Roche), the cap analogue G(ppp)G (f.c. 0.33 mM; Prof. Edward Darzynkiewicz, Warsaw University) and ^{14}C-labeled leucine (11–35 μM, depending on the individual experiment) (PerkinElmer), filling up the volume with water to reach a final volume of 1000 μl. The addition of the caspase inhibitor Z-VAD-FMK (benzyloxycarbonyl-Val-Ala-Asp(OMe)-fluoromethylketone) (30 μM; Promega) was optional in batch and CECF reactions. Z-VAD-FMK is reported to be a general caspase inhibitor inhibiting the 14 members of the caspase family known today [41]. In order to inhibit bacterial growth during prolonged incubation times, sodium azide was added to the translation reaction and the feeding mixture at a final concentration of 0.02%. In order to evaluate optimal conditions for disulfide bond formation *in vitro*, the translation buffer was supplemented with 0.5 mM GSH and 2.5 mM GSSG or 2.5 mM DTT. After

filling the chambers with reaction and feeding mixture, they were sealed with a plastic foil. Dialysis reactions were incubated at 27°C in a thermomixer (Thermomixer comfort, Eppendorf) for up to 48 h with gentle agitation at 600 rpm. The thermomixer itself was placed in an incubator heated to 28°C in order to avoid condensation of water. Background translational activity in insect lysates was monitored by incubating a translation mixture without the addition of a DNA template (= no template control, NTC). In order to separate insect vesicles from the translation mixture a centrifugation step (16,000×g, 10 min, 4°C) was performed at the end of incubation. After separating the supernatant from pelleted insect vesicles, the vesicular fraction was resuspended in phosphate-buffered saline (PBS).

Quantification of cell-free expressed and ^{14}C-leucine labeled proteins

At the indicated incubation time aliquots of 5 µl were withdrawn from the cell-free translation reaction, mixed with 3 ml trichloroacetic acid and incubated in a 80°C water bath for 15 min, followed by incubation on ice for 30 min. In order to remove non-incorporated ^{14}C-leucine from the translation mixture, protein solutions were filtered using a vacuum filtration system (Hoefer). Incorporation of ^{14}C-leucine in cell-free expressed proteins was measured by liquid scintillation counting using the LS6500 Multi-Purpose scintillation counter (Beckman Coulter).

SDS-PAGE and autoradiography

SDS-PAGE was performed using precast gels (NuPAGE, 10% Bis-Tris, Life technologies). Aliquots of the translation mixture, the supernatant and the vesicular fraction (5 µl) were precipitated in cold acetone and left on ice for at least 15 min. Samples were centrifuged at 16,000×g and protein pellets were dried for 1 h at 45°C. Dried protein pellets were resuspended in 20 µl of 1× sample buffer (NuPAGE LDS Sample Buffer supplemented with 50 mM DTT, Life technologies) and incubated for 15 min at room temperature on an orbital shaker. Samples were loaded onto precast SDS-PAGE gels and run at 200 V for 35 min. For quality control gels were stained with Coomassie Blue (SimplyBlue SafeStain, Life technologies). Stained gels were subsequently dried on Whatman paper for 60 min at 70°C (Unigeldryer 3545D, Uniequip). Radioactively labeled proteins were visualized using a phosphorimager system (Typhoon TRIO + Imager, GE Healthcare). Deglycosylation of Mel-EPO and Mel-vtPA was performed using peptide-N-glycosidase F (PNGase F) (NEB). Aliquots of the translation mixture (5 µl) were treated with PNGase F according to the manufacturer's instructions and samples were analyzed by SDS-PAGE and autoradiography.

Analysis of fluorescent fusion proteins

Cell-free synthesized fluorescent fusion proteins (eYFP, Mel-Hb-EGF-eYFP) were analyzed quantitatively in a phosphorimager system (Excitation 488 nm, emission filter 526 nm short-pass; Typhoon TRIO + Imager, GE Healthcare) and qualitatively by confocal laser scanning microscopy using a LSM 510 Meta (Zeiss). Complete translation mixtures, the supernatant and vesicular fractions were diluted 1:3 (qualitative analysis) or 1:6 (quantitative analysis) in PBS and transferred to Ibidi slides (µ-Slide, 18 well, Ibidi). For confocal microscopy samples were excited at 488 nm using an argon laser and emission signals were recorded using a bandpass filter in the wave length range of 505 nm to 550 nm. Prior to photobleaching, samples of Mel-Hb-EGF-eYFP were diluted 1:4 in the supernatant of the corresponding reaction mixture in order to maintain the same concentration of non-translocated Mel-Hb-EGF-eYFP and endogenous lysate proteins and to dilute the amount of insect vesicles. Translation mixtures of cell-free synthesized eYFP were treated likewise. Photobleaching was performed using an argon laser at 488 nm with 100% laser intensity and 500 iterations.

Preparation of fibrin-agarose-plates

In order to analyze the biological activity of cell-free produced Mel-vtPA, fibrin-agarose-plates were prepared according to Granelli-Piperno and Reich [42] with minor modifications. First, three solutions a, b, c were prepared separately from each other. Solution a: 2.5% low-melting agarose (AppliChem) was boiled for 2 min and tempered at 42°C. Solution b: Thrombin (Merck) and Plasminogen (Merck) were diluted in preheated PBS (37°C) to final concentrations of 18 µg/ml and 0.2 U/ml, respectively. Solution c: Fibrinogen (Merck) was diluted in preheated PBS (37°C) to yield a final concentration of 10 mg/ml. Solution a (3.2 ml) was mixed with 1.9 ml of solution b, followed by addition of 1.3 ml solution c. The resulting mixture was poured evenly into a petri dish (Kuhnle, size 12×12 cm). Cleavage of fibrinogen to fibrin by thrombin subsequently leads to the formation of the fibrin network. Plates were stored at 4°C until usage.

Analysis of Mel-vtPA activity

Tissue-type plasminogen activators are important thrombolytic agents. They belong to the class of serine proteases and convert the inactive proenzyme plasminogen to active plasmin. Plasmin itself solubilizes polymerized fibrin networks into soluble products leading to the degradation of blood clots [40]. To analyze the biological activity of cell-free produced Mel-vtPA, aliquots of the translation mixture were treated with the mild detergent n-dodecyl-β-maltoside (DDM) (final concentration of DDM = 0.1%) in order to release translocated target proteins from the lumen of the insect vesicles. Then, samples were agitated for 45 min at room temperature. Protein yields of radioactively labeled Mel-vtPA were determined as described above. Samples were diluted to a final concentration of 0.5 µg/ml Mel-vtPA. Aliquots of 1 µl were pipetted onto the fibrin-agarose-plate and incubated at 37°C for 24 h. Depending on the activity of cell-free produced tPA initial lytic zones became visible after an incubation time of 2 h to 4 h. Lytic zones were measured and evaluated after 24 h of incubation.

Results

Expression of eYFP in the insect CECF system

In this study, a small-scale dialysis device was used, consisting of 50 µl reaction chamber and a 1000 µl feeding chamber, separated by semi-permeable dialysis membrane. Protein synthesis takes place in the reaction mixture, being supported by the continuous supply of substrate and energy components and the continuous removal of inhibitory reaction by-products, both diffusing through the membrane. In order to investigate if the vesicle-containing eukaryotic cell lysate could be applied in this dialysis system, we first evaluated the expression of the fluorescent and soluble protein eYFP. Cell-free expression of eYFP in insect lysate was performed using the batch and CECF mode over a total incubation time of 24 h. Translation efficiency was evaluated by the measurement of protein fluorescence intensity in translation reactions. These initial experiments resulted in a twofold increase in fluorescence intensity of eYFP synthesized in the insect CECF mode compared to the batch mode after 24 h of incubation (data not shown). Due to these positive results we further improved the system's synthesis efficiency. In batch reactions we have seen that prolonged

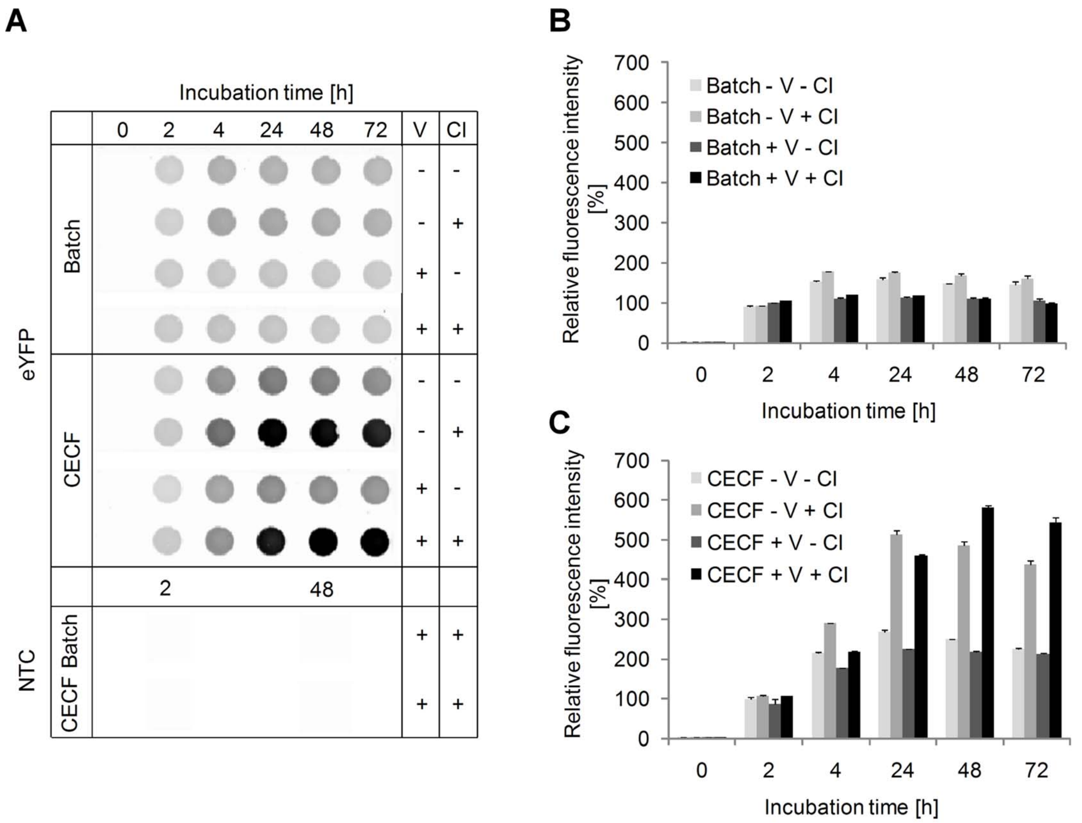

Figure 1. Time course for the cell-free expression of eYFP. Reactions were carried out using an *in vitro* translation system based on insect lysates in batch and CECF mode in the presence (+) and absence (−) of insect vesicles (V) and caspase inhibitor (CI). A) Fluorescence imaging of eYFP using a phosphorimager system. B) Relative fluorescence intensity of eYFP in batch reactions. C) Relative fluorescence intensity of eYFP in CECF reactions. The percentage calculation of the fluorescence intensity is depicted by the fluorescence intensity of eYFP measured after 2 h of incubation set as 100% (batch, + V, - CI). For each data point fluorescence intensity of eYFP is presented as mean value of duplicate analysis, with the error bar indicating value 1 and value 2 of the duplicate. NTC = No template control; translation reaction without addition of a DNA template.
doi:10.1371/journal.pone.0096635.g001

incubation times may lead to the proteolytic degradation of *de novo* synthesized target proteins, such as the human epidermal growth factor receptor. The proteolytic degradation of target proteins in cell-free systems can be efficiently inhibited by the addition of protease inhibitors, as has been shown before for the synthesis of Fab fragments in an *E. coli*-based cell-free expression system [43]. Likewise it was reported that the addition of the caspase inhibitor YVAD-CMK (N-acetyl-Tyr-Val-Ala-Asp-chloromethylketone) to cell extracts derived from chicken DU249 cells blocked apoptotic events such as DNA fragmentation and proteolysis [44]. Another caspase inhibitor, Z-VAD-FMK, was reported to inhibit apoptosis efficiently in *Sf*9 cells, *Sf*21 cells and *Drosophila melanogaster* S2 cells [45,46]. The cell-free extract applied in this study is prepared from *Sf*21 insect cells, thus we decided to evaluate different protease and caspase inhibitors, in order to inhibit the proteolytic degradation of target proteins. In this context, we found that the addition of the general caspase inhibitor Z-VAD-FMK showed a positive effect on the preservation and stability of selected target proteins in the translation mixture after prolonged incubation times (Figure S1). Interestingly, this protection was not achieved when a commercially available broad-spectrum protease inhibitor cocktail was added to the translation mixture, indicating the specific influence of caspases. Thus, we tested the caspase inhibitor likewise in the insect CECF system. Both batch and CECF reactions were performed in the presence and absence of the caspase inhibitor. Furthermore, eYFP was expressed in insect lysate containing endoplasmic reticulum-derived vesicles as well as in lysate which was depleted of endogenous vesicles prior to its use in the cell-free reaction. Batch and CECF reactions were compared regarding their fluorescence intensity of *de novo* synthesized eYFP. Additionally, total protein yields were determined by hot TCA precipitation and subsequent scintillation counting. Both parameters were normalized to the values obtained for eYFP synthesized in a 2 h-standard batch reaction (= 100%) in presence of insect vesicles (+ V) and in absence of caspase inhibitor (- CI). As expected, batch reactions of eYFP reached the plateau phase after 2 h (protein quantification) to 4 h (fluorescence analysis) of incubation. In batch reactions, the presence or absence of caspase inhibitor and insect vesicles did not show any significant influence on the fluorescence intensity and on the total protein yield of eYFP (Figure 1, Figure S2). In contrast, the addition of caspase inhibitor to CECF reactions significantly prolonged the reaction life time from 2 h to 48 h. Maximum fluorescence intensity of eYFP was detected in the CECF reaction after 48 h of incubation in presence of insect vesicles and caspase inhibitor. Analysis of this CECF reaction revealed a more than fivefold increase in fluorescence intensity and more than fourfold increase in total protein yields compared to standard batch reactions (Figure 1, Figure S2). Moreover, we were able to show that the vesicle-containing insect lysate is absolutely compatible with the applied dialysis membrane and device, since no negative influence on the synthesis efficiency, e.g. caused by clogging of the dialysis membrane, was observed.

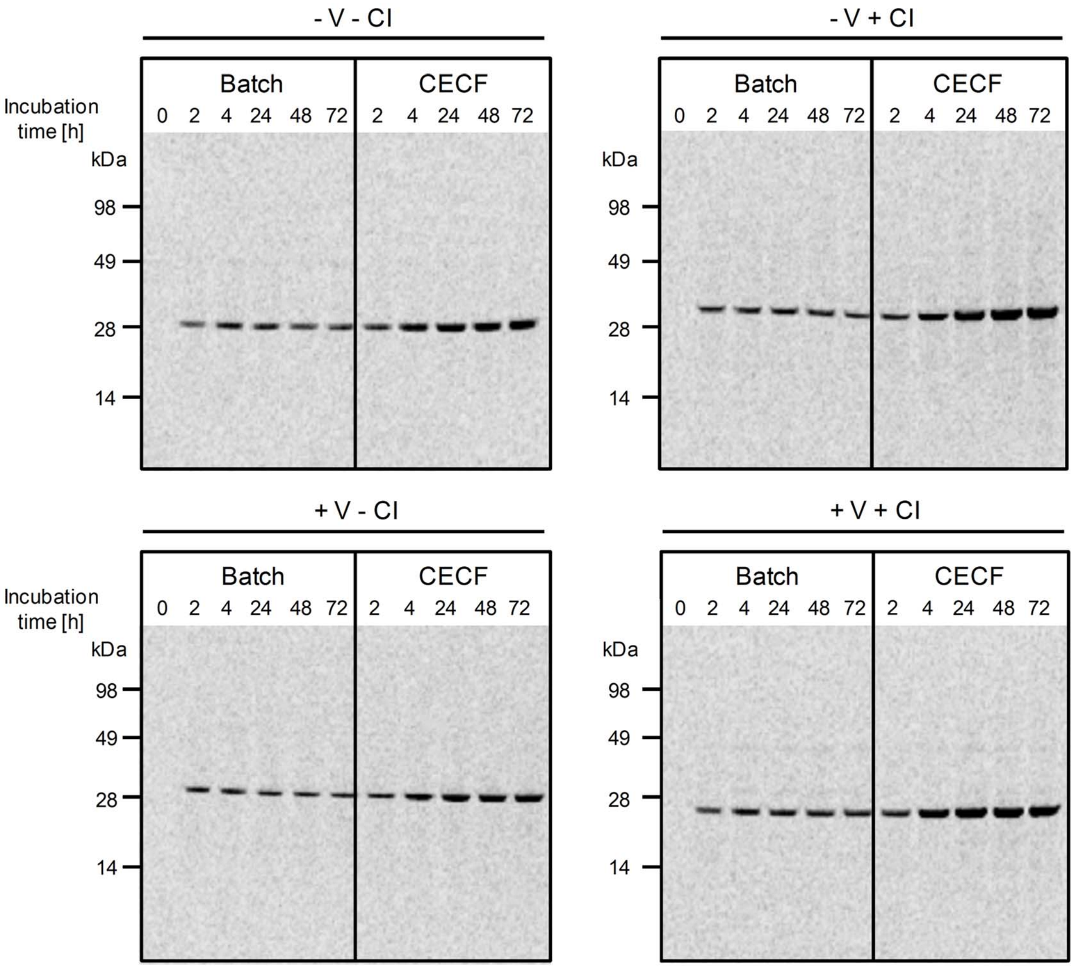

Figure 2. Time course of ^{14}C-leucine labeled eYFP synthesized in batch and CECF mode. Cell-free reactions using insect lysate were carried out in the presence (+) and absence (−) of insect vesicles (V) and caspase inhibitor (CI). Translation mixtures were analyzed by SDS-PAGE and autoradiography. Cell-free synthesized eYFP shows a migration pattern corresponding to its expected molecular mass (calculated molecular mass = 29 kDa).
doi:10.1371/journal.pone.0096635.g002

Analysis of cell-free synthesized eYFP by SDS-PAGE and autoradiography confirmed size and integrity of the target protein in all reactions (Figure 2). The most prominent protein bands of eYFP were observed in CECF reactions supplemented with caspase inhibitor, confirming the results obtained by fluorescence analysis and protein quantification. Fractionation of all samples into supernatant and vesicular fractions revealed that cell-free synthesized eYFP was soluble and could be transferred and detected in the supernatant (Figure S3).

Influence of caspase inhibitor on the expression of different model proteins using the insect CECF system

To evaluate the performance of the insect cell-free expression system, we have chosen a set of representative model proteins, among them the cytosolic protein eYFP, the transmembrane proteins Mel-Hb-EGF-eYFP, endothelin-B receptor and bacteriorhodopsin. Furthermore, test candidates were complemented by the human glycoproteins Mel-EPO and Mel-vtPA. Model proteins were expressed in the coupled insect cell-free batch system as well as in an *E. coli*-based batch cell-free translation system (EasyXpress Protein Synthesis Kit, Qiagen). All chosen model proteins were expressed in the insect cell-free expression system, whereas in *E. coli* three out of seven proteins failed to be expressed at detectable levels (Figure S4). In the case of prokaryotic bacteriorhodopsin this observation was explained by the fact that the DNA template of bacteriorhodopsin was codon-optimized for expression in *Sf*21 cells (GeneArt Gene Synthesis, Life technologies). Next, we examined the expression of the chosen model proteins using the insect CECF system. Cell-free protein synthesis was performed for 48 h in presence and absence of caspase inhibitor. For all tested target proteins lowest protein yields were achieved using the insect batch system with and without caspase inhibitor (Figure 3 a). In comparison to batch-based reactions, an approximate twofold increase in protein yield was achieved by applying the insect CECF system without caspase inhibitor. The highest protein yields were observed using the insect CECF system supplemented with caspase inhibitor. The comparison of standard batch reactions with CECF reactions supplemented with caspase inhibitor demonstrated an average increase in protein yield of more than four- to fivefold depending on the individual protein. Protein quantification data was supported by the qualitative analysis of translation mixtures by fluorescence imaging (Figure 3 b) and autoradiography (Figure 3 c). The strongest protein bands were observed at the expected size in CECF reactions performed in presence of caspase inhibitor. In the case of Mel-EPO four distinct

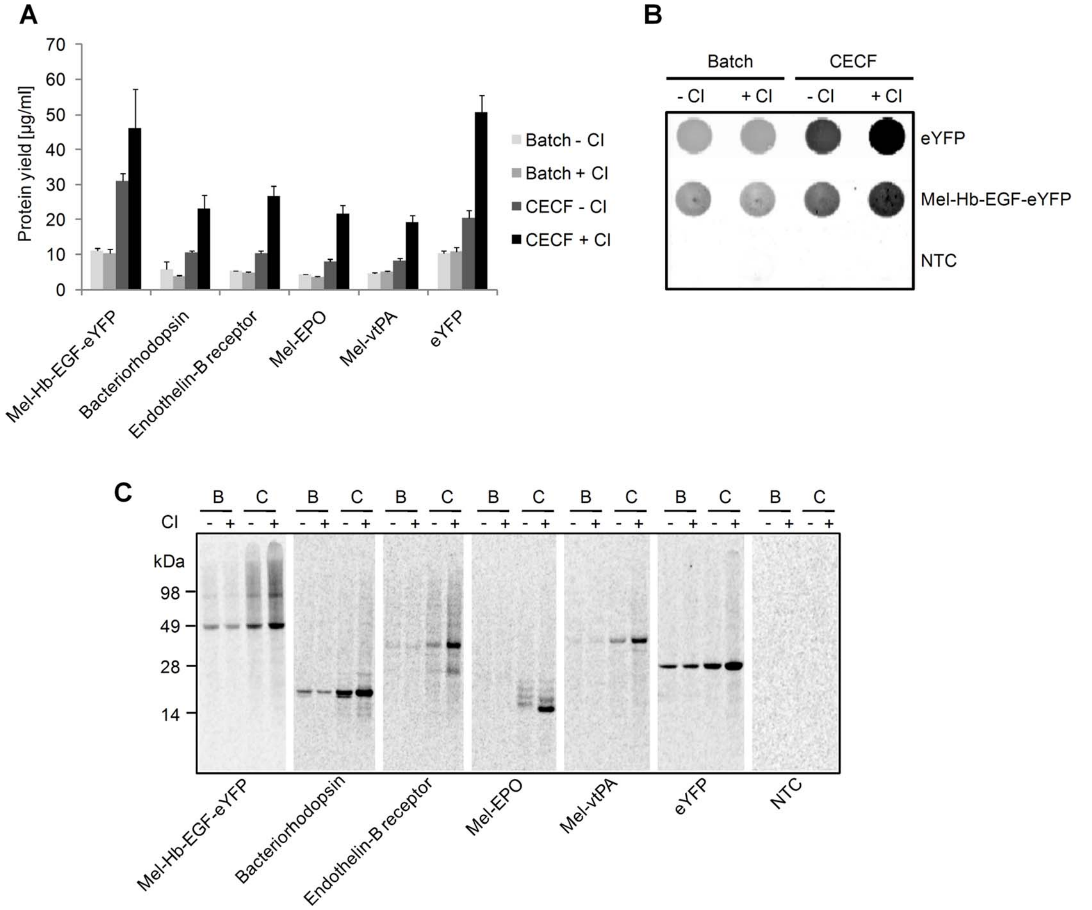

Figure 3. Influence of caspase inhibitor (CI) on the expression yield of representative model proteins. The membrane proteins Mel-Hb-EGF-eYFP (51 kDa), bacteriorhodopsin (27 kDa) and endothelin-B receptor (49 kDa), the glycoproteins Mel-EPO (21 kDa, protein in its non-glycosylated form) and Mel-vtPA (41 kDa, protein in its non-glycosylated form) as well as the protein eYFP (29 kDa) were synthesized in batch (B) and CECF (C) reactions in the presence (+) and absence (−) of CI using insect lysate. Reactions were carried out for 48 h in the absence of the reducing agent DTT and in presence of ^{14}C-leucine. A) Diagram showing total protein yields which were determined by incorporation of ^{14}C-leucine and liquid scintillation counting. Standard deviations were calculated from triplicate analysis (n = 3). B) Fluorescence imaging of eYFP and Mel-Hb-EGF-eYFP using a phosphorimager system. NTC = No template control; translation reaction without addition of a DNA template. C) Qualitative analysis of cell-free synthesized proteins by SDS-PAGE and autoradiography. Addition of CI significantly increased the protein yield of all target proteins analyzed in this study.
doi:10.1371/journal.pone.0096635.g003

protein bands were observed. Digestion of Mel-EPO with PNGase F resulted in one single protein band showing a migration pattern which matches the expected molecular mass of non-glycosylated Mel-EPO. With respect to this observation it can be assumed that the lowest protein band corresponds to the protein in its non-glycosylated form, whereas the upper three protein bands correspond to glycosylated species of Mel-EPO (deglycosylation assay of Mel-EPO see Figure S5).

Time course of Mel-Hb-EGF-eYFP in batch and CECF reactions

The results presented demonstrate that total yields of cell-free synthesized membrane proteins can be significantly increased by using the eukaryotic CECF system instead of the conventional batch-based system. In order to analyze the accumulation of membrane proteins in the vesicular fraction of the cell lysate, a time course expression of Mel-Hb-EGF-eYFP was performed.

The protein was synthesized in batch and CECF mode over a time range of 48 h in the presence of caspase inhibitor. After defined incubation times (0 h, 2 h, 4 h, 24 h, 48 h) translation was stopped by lowering the temperature to 4°C and samples were fractionated into the supernatant and the vesicular fraction. Batch reactions reached their maximum yield of Mel-Hb-EGF-eYFP after 2 h of incubation with a total yield of *de novo* synthesized membrane protein of 20 µg/ml in the translation mixture and 9 µg/ml in the vesicular fraction (Figure 4 a). CECF reactions reached their plateau phase after 24 h of incubation yielding 57 µg/ml target protein in the translation mixture (almost threefold increase when compared to batch-based reactions) and 32 µg/ml in the vesicular fraction (almost fourfold increase when compared to batch-based reactions) (Figure 4 b). These quantitative measurements were supported by data obtained from autoradiography. In batch reactions a minor decrease in protein yield was observed from 4 h until 48 h of incubation (Figure 4 c). In contrast, protein bands of CECF reactions gained intensity over time which accounted for the complete translation mixture as well as for the vesicular fraction (Figure 4 d).

Protein quantification by TCA precipitation and liquid scintillation counting does not allow the discrimination between insoluble aggregates of membrane proteins and correctly folded

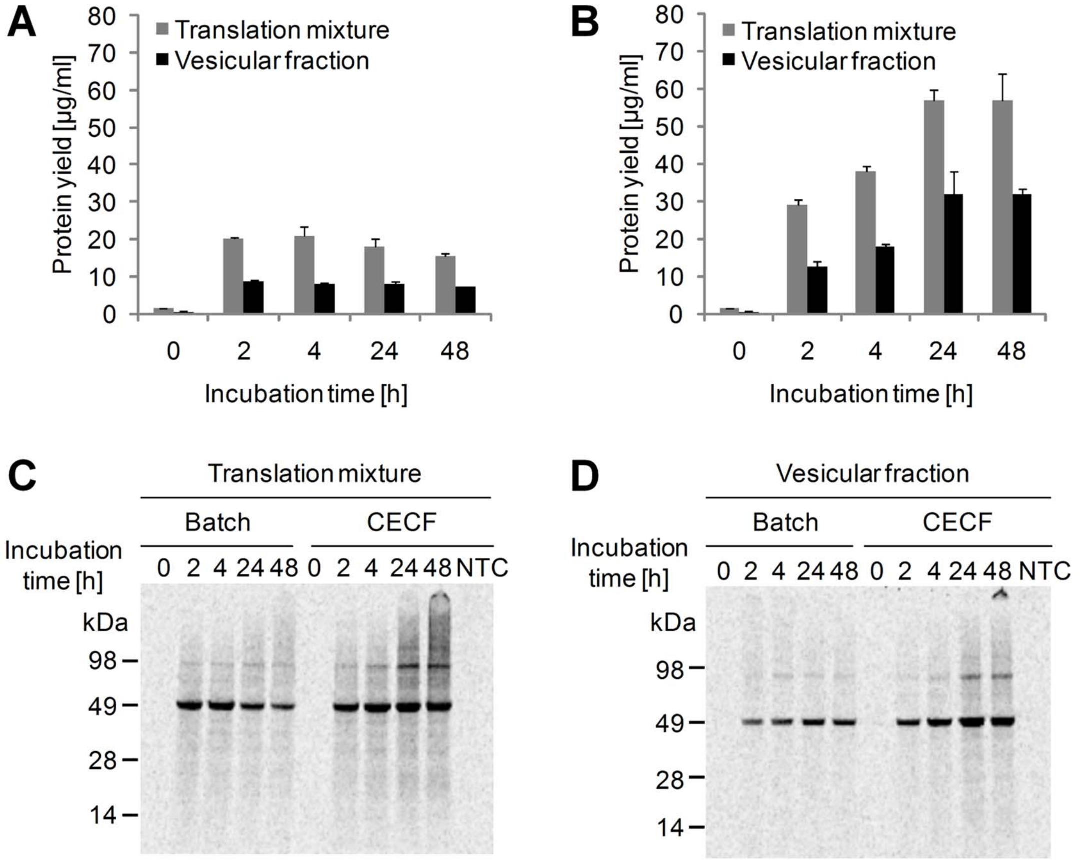

Figure 4. Time course analysis of the cell-free synthesized type-I transmembrane protein Mel-Hb-EGF-eYFP. Batch and CECF reactions were carried out in the presence of caspase inhibitor and ^{14}C-leucine and in the absence of DTT. Protein yields in batch (A) and CECF reactions (B) were determined in the translation mixture and the vesicular fraction by liquid scintillation counting. Standard deviations were calculated from triplicate analysis (n = 3). C) Qualitative analysis of Mel-Hb-EGF-eYFP in the translation mixture by SDS-PAGE and autoradiography. D) Analysis of Mel-Hb-EGF-eYFP in the vesicular fraction. Cell-free synthesized Mel-Hb-EGF-eYFP shows a migration pattern corresponding to its expected molecular mass (calculated molecular mass = 51 kDa). NTC = No template control; translation reaction without addition of a DNA template.
doi:10.1371/journal.pone.0096635.g004

membrane proteins, which are inserted into the lipid bilayer of the insect vesicles. In both cases, proteins are pelleted during the centrifugation step. In the case of an efficient embedding of Mel-Hb-EGF-eYFP in insect vesicles, fluorescent proteins should be detectable in the vesicular membrane using confocal microscopy. Cell-free expression of Mel-Hb-EGF-eYFP was performed for 2 h in batch and 24 h in CECF mode in the presence of caspase inhibitor. Confocal images show the strongest fluorescence intensity for Mel-Hb-EGF-eYFP synthesized in the CECF reaction (Figure 5 a), demonstrating the potential of the eukaryotic CECF system to enrich a certain membrane protein in vesicles with higher protein yields compared to the conventional batch system. In order to analyze the localization of Mel-Hb-EGF-eYFP in the insect lysate, translation mixtures were fractionated into the supernatant and the vesicular fraction. As expected, the membrane protein showed strong fluorescence emission in the translation mixture and the vesicular fraction but not in the supernatant (Figure 5 b).

Fluorescence recovery after photobleaching (FRAP) experiments were performed in order to investigate the embedding of *de novo* synthesized membrane proteins into the vesicular membrane of insect microsomes. Samples of Mel-Hb-EGF-eYFP and eYFP were analyzed before and after extensive photobleaching. The endogenous vesicles of the insect lysate display a size ranging from approximately 1 to 5 µm. Confocal images were taken from vesicle aggregates which were surrounded by the cytosolic fraction of the lysate. Fluorescent vesicles harboring Mel-Hb-EGF-eYFP were bleached completely after 500 iterations with 100% laser intensity. According to our expectations, no fluorescence recovery was observed after 50 s of incubation which shows that the fluorescent target protein was not delivered passively by diffusion from the cytosolic surrounding (Figure 5 c). As a control, samples of eYFP were treated likewise. As expected, the laser exposure did not result in a detectable photobleaching effect of the cytosolic protein eYFP (Figure S6).

Synthesis and functional analysis of Mel-vtPA

Disulfide bond formation is an important posttranslational modification in proteins, exhibiting a major influence on protein stability, folding and functionality [47,48,49,50]. In order to monitor disulfide bond formation in cell-free synthesized proteins, we have chosen Mel-vtPA as a model protein, which consists of two structural domains and contains nine intramolecularly formed disulfide bonds. In the case of vtPA, formation of protein domains that are stabilized by disulfide bonds and biological activity are tightly connected [51]. In order to optimize the insect cell-free translation system for the expression of functional disulfide-bonded proteins, we have evaluated different insect lysate batches and different translation buffers.

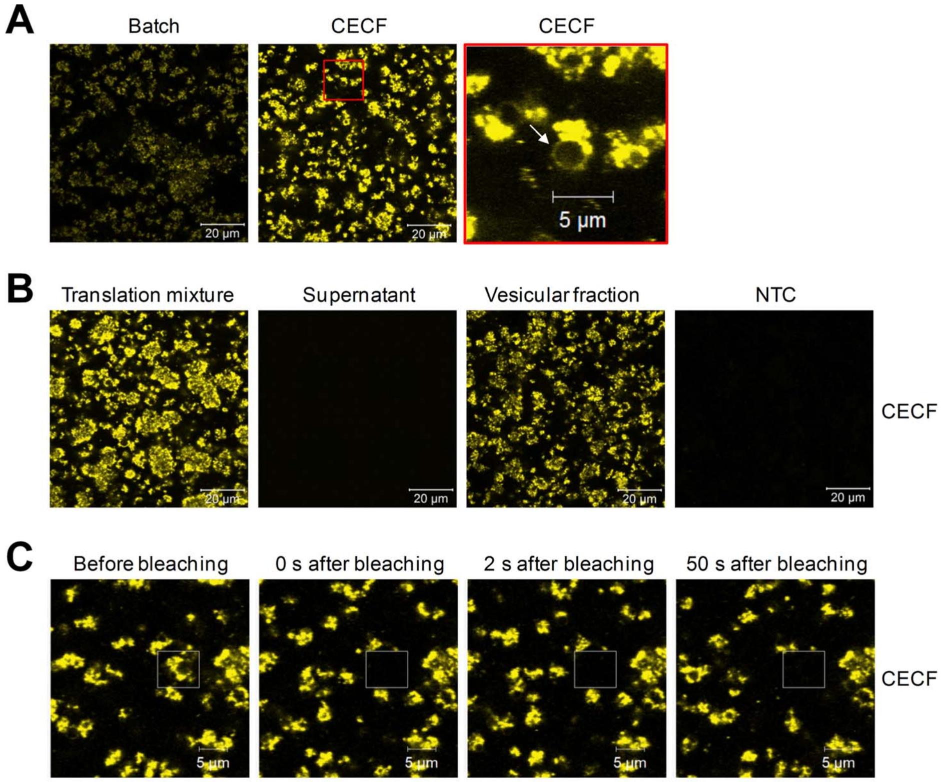

Figure 5. Confocal images of cell-free synthesized Mel-Hb-EGF-eYFP. Mel-Hb-EGF-eYFP was synthesized in batch mode for 2 h and CECF mode for 24 h in presence of caspase inhibitor. A) Direct comparison of batch and CECF reactions using identical laser and detector settings. The red-framed picture at the far right is showing the magnification of an inset from the centered picture. The arrow indicates a vesicular membrane harboring cell-free synthesized Mel-Hb-EGF-eYFP. B) Fluorescent Mel-Hb-EGF-eYFP was analyzed in the translation mixture, the supernatant and the vesicular fraction. Strong emission intensity of *de novo* synthesized target proteins was observed in microsomal structures in the translation mixture and the vesicular fraction. NTC = No template control; translation reaction without DNA template. C) Photobleaching of Mel-Hb-EGF-eYFP present in the translation mixture. No fluorescence recovery of Mel-Hb-EGF-eYFP was observed after 50 s of incubation indicating that fluorescent and membrane-embedded Mel-Hb-EGF-eYFP was not delivered by diffusion from the cytosolic surrounding.
doi:10.1371/journal.pone.0096635.g005

First, three translation buffers [(1) buffer containing DTT; (2) buffer without DTT and (3) buffer without DTT, containing GSH and GSSG] were tested in combination with insect lysate supplemented with GSH and GSSG. Functional Mel-vtPA was expressed in all three mixes, but – in accordance to our expectations - the presence of the reducing agent DTT in the reaction mixture negatively affected the functionality of the target protein (Figure S7). Next, we examined the influence of differently prepared insect lysate batches in combination with a DTT-deficient translation buffer on the functionality of cell-free synthesized Mel-vtPA. Cell-free reactions were performed in batch and CECF mode in presence of caspase inhibitor and ^{14}C-leucine using three differently composed reaction mixtures: (i) lysate without GSH and GSSG; (ii) lysate supplemented with GSH and GSSG and (iii) buffer and lysate supplemented with GSH and GSSG. Regarding total protein yields, no significant differences were observed between the three translation mixtures using the batch mode (Figure 6 a). In contrast, for CECF reactions protein quantification data revealed distinct differences between the three translation mixtures. Using the CECF system, highest protein yields were determined for Mel-vtPA using the translation buffer without GSH and GSSG in combination with insect lysate supplemented with GSH and GSSG (maximum yield = 18 µg/ml). Functional Mel-vtPA was produced in the batch and CECF system using the three differently composed translation mixtures (Figure 6 b, c). The activity of cell-free produced Mel-vtPA was comparable to a commercially available positive control (recombinant full length tPA, Anaspec) which was tested in parallel, indicating that our optimized insect cell-free expression system efficiently produces complex, biologically active disulfide-bonded proteins (Figure 6 d).

Discussion

Since it is well-known that batch reactions have only short reaction life times and thus limited protein yields, we aimed to combine the well-established CECF format [33,35] with our eukaryotic insect lysate. Application of the insect CECF system resulted in significantly increased protein yields compared to batch reactions. In this study, the reaction compartment of the chosen CECF device and the analyzed batch reactions comprised the same volume (50 µl), and thus both required the same volume of insect lysate. As the CECF system reaches higher protein yields, one can conclude that the same volume of lysate can be more efficiently used in the insect CECF system compared to the batch system.

On the other hand, insect CECF reactions depend on the preparation of a 1 ml feeding solution, and thus reagent costs are naturally increased compared to insect batch reactions. At the current state, increase in protein yield does not correlate with increase in costs. This is an issue that needs to be improved in the future. One possibility to increase the production yields in cell-free

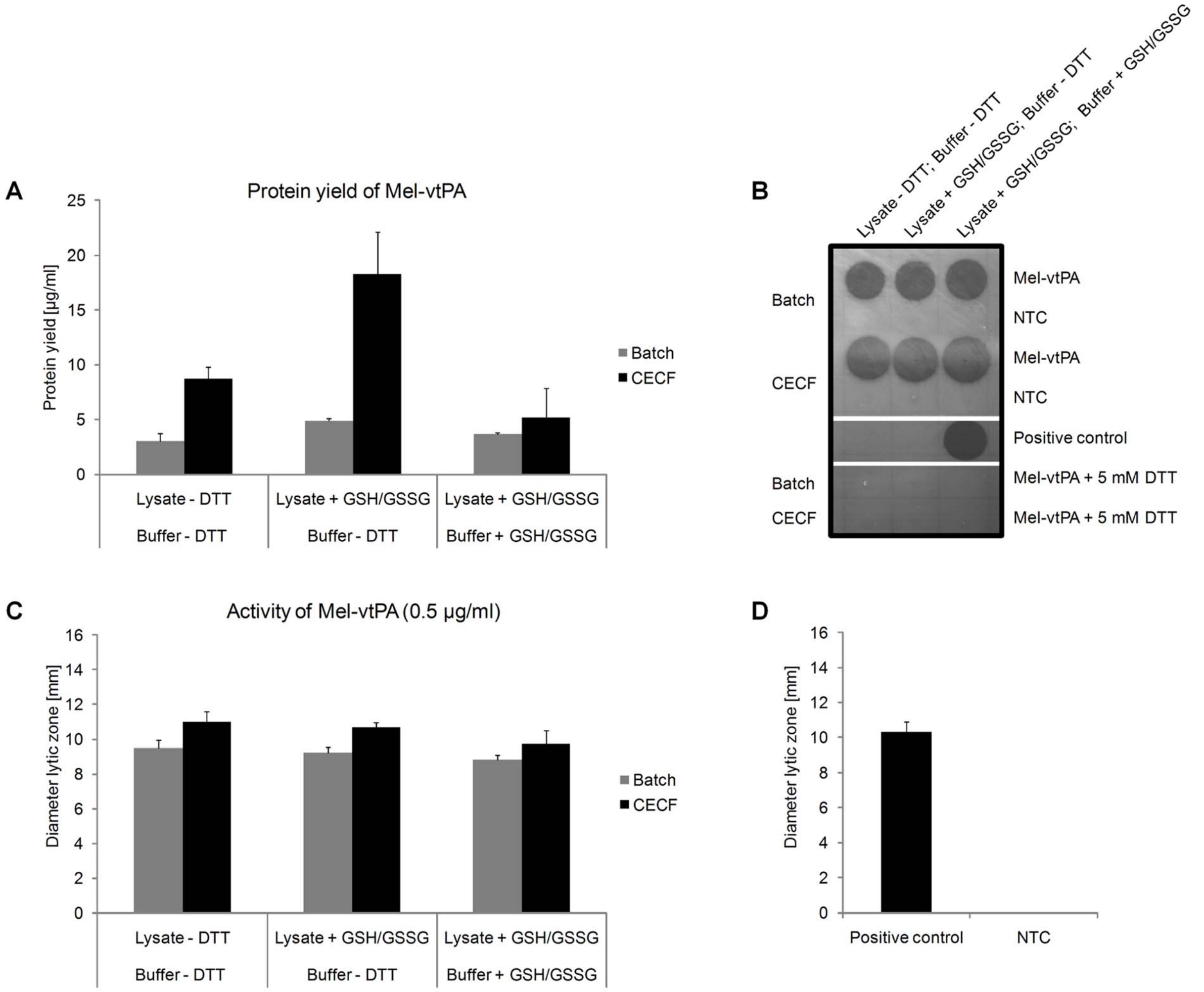

Figure 6. Analysis of Mel-vtPA expression and activity. Cell-free reactions were performed for 48 h in batch and CECF mode in absence of DTT, but in presence of caspase inhibitor and ^{14}C-leucine using three differently composed reaction mixtures. (i) lysate without GSH and GSSG; (ii) lysate supplemented with GSH and GSSG and (iii) buffer and lysate supplemented with GSH and GSSG. A) Diagram showing total yields of ^{14}C-leucine labeled Mel-vtPA. B) Fibrin-agarose-plate showing lytic zones created by biologically active Mel-vtPA. All samples were diluted to 0.5 µg/ml. Activity of Mel-vtPA is completely abolished after addition of 5 mM DTT. C) Activity of Mel-vtPA analyzed by fibrin-agarose-plate assay. D) Activity of the positive control (= purified full length tPA, 0.5 µg/ml, Anaspec) analyzed by fibrin-agarose-plate assay. NTC = No template control; translation reaction without addition of a DNA template. Standard deviations were calculated from triplicate analysis (n = 3).
doi:10.1371/journal.pone.0096635.g006

systems might be the switch from cap-dependent to cap-independent translation initiation, since translation initiation is known to be the rate-limiting step in protein synthesis [52,53]. This strategy has been successfully employed for batch-based translation systems based on CHO and human cell extracts, by using advanced DNA templates bearing an internal ribosome entry site (IRES) in the 5′ untranslated region of the target gene [18,19]. However, this publication states the proof-of-concept that lysates which contain endogenous microsomal vesicles are absolutely compatible with classical dialysis systems, thus paving the way for establishing novel eukaryotic *in vitro* translation systems with improved properties.

In this study, we additionally investigated the influence of the general caspase inhibitor Z-VAD-FMK on the protein synthesis performance using the insect cell-free translation system. In previous studies it has been shown that Z-VAD-FMK inhibits apoptosis efficiently in *Sf*9 cells, *Sf*21 cells and *Drosophila melanogaster* S2 cells [45,46]. Here, we demonstrate the positive effect of Z-VAD-FMK on protein synthesis efficiency using a eukaryotic *in vitro* translation system based on *Sf*21 cells. The addition of Z-VAD-FMK to cell-free reactions led to significantly prolonged reaction life times and increased overall protein yields. In addition to Z-VAD-FMK, we have also tested other inhibitor types, among them inhibitors with varying peptides (e.g. Asp-Glu-Val-Asp, DEVD) and chemical groups (chloromethylketone as well as aldehyde groups). Likewise, we noticed the positive effect of these inhibitors on the overall protein yield (data not shown).

Caspases (cysteine proteases with aspartate-specificity) are key regulators of apoptosis, also termed as programmed cell death. On the basis of our results we assume that caspases may be activated during the cell lysate preparation procedure and/or during the translation reaction itself, in particular after prolonged incubation times. This activation may then lead to the degradation of endogenous lysate proteins, such as translation factors, and also *de novo* synthesized target proteins. From previous studies it is known that caspases are involved in the cleavage of translation initiation factors, such as eIF4G [54,55,56], eIF4B, eIF4E-binding protein (eIF4E-BP1) [57], eIF2α [58], and eIF3j [57], a process which

results in the inhibition of protein synthesis. In this context, it was reported that the cleavage of translation initiation factor eIF2α was completely inhibited in HeLa cells pretreated with caspase inhibitor Z-VAD-FMK [59]. Taking these observations into account, the positive effect of Z-VAD-FMK on total protein yields using the insect CECF system could be potentially explained by the preservation of translation initiation factors over prolonged incubation times, which, as a result, may cause a gain in translation efficiency.

The insect cell-free system used in this study enables the synthesis of correctly folded and biologically active proteins with multiple disulfide bonds. In contrast to other systems, it does not require a chemical pretreatment of the cell extract or the addition of exogenous proteins in order to yield soluble and functional disulfide-bonded proteins [11,60,61,62]. As demonstrated in previous studies, for the expression of functional scFv antibody and Fab fragments [30,31], solely the adjustment of redox conditions is sufficient to stabilize the redox potential. The positive effect of glutathione has been shown before for a GroEL-GroES-enriched *E. coli*-based cell-free translation system, where functional *Candida antarctica* lipase B, a protein requiring three disulfide bonds to be active, has been produced by adding the appropriate amount of oxidized glutathione [63].

tPA, one of the model proteins used in this study, is of important clinical relevance, since it is involved in the thrombolysis of blood clots, tissue remodeling and cell migration [51]. Production of tPA is challenging and cost-intensive [64]. So far, tPA has been recombinantly expressed *in vivo* [65,66,67] as well as *in vitro* using cell extracts from rabbit reticulocytes [39] and *E. coli* cells [38,68]. However, expression of tPA *in vivo* requires complicated purification processes and in the case of expression in *E. coli*, normally *in vitro* refolding is required [68] as well as elaborate downstream purification processes in order to remove endotoxins [69]. tPA which has been produced in eukaryotic cell lysates is naturally free of endotoxins, thus subsequent downstream purification processes are considered to be less difficult compared to purification of products from *E. coli* cell extracts (Chen, 2009). Here, we demonstrate the synthesis of active Mel-vtPA in a eukaryotic cell-free translation system at reasonable yields (~20 µg/ml) for functional studies in only one to two days at costs that the average research lab can afford. Even without purification, proteins synthesized in the insect CECF system could potentially be applied directly in cell-based assays. Of course, if the product is destined to become a mammalian injectable purification is preferred, since endogenous proteins contained in the insect lysate probably cause immune reactions, which may not be acceptable from the regulatory point of view [70]. On the other hand, for the production of vaccines this effect was shown to stimulate autoantibody production against metastatic melanomas in mice, thus facilitating an "adjuvant effect" of insect cells [71].

CECF-based cell-free translation systems using *E. coli* lysates and wheat germ extracts are well-established and commercially available (e.g. RTS 500 ProteoMaster *E. coli* HY Kit and RTS 500 Wheat Germ CECF Kit, 5PRIME). These systems are highly productive and enable the synthesis of selected target proteins in amounts sufficient for further functional and structural studies, e.g. by NMR spectroscopy and X-ray crystallography [72,73]. Nevertheless, the insect CECF system represents a promising alternative to already established systems, since it enables the incorporation of eukaryotic co-and posttranslational modifications into cell-free synthesized proteins [13,14]. Due to this ability, time course experiments could provide interesting insights in the posttranslational modification, e.g. glycosylation pattern, of *de novo* synthesized target proteins over prolonged incubation times. Moreover, the endogenous vesicles of the eukaryotic lysate are an important prerequisite for the embedding of membrane proteins into a natural lipid bilayer, circumventing the potential toxicity of membrane proteins when overexpressed in living cells. The expression of complex and high-molecular mass membrane proteins, in particular facilitated by the eukaryotic CECF system presented in this study, constitutes a basis for the production of sufficient amounts of correctly folded and functional membrane protein samples for further characterization by structural and functional studies.

Supporting Information

Figure S1 Influence of caspase inhibitor on the stability of *de novo* synthesized membrane proteins. Cell-free synthesis of the epidermal growth factor receptor (fused to a melittin signal peptide at the N-Terminus and eYFP at the C-Terminus, 163 kDa) was performed using the coupled insect cell-free system in batch mode in presence of ^{14}C-leucine and in presence and absence of caspase inhibitor Z-VAD-FMK. Reactions were stopped at the indicated incubation times. In addition, one reaction was performed in presence of a commercially available protease inhibitor mix ("Complete protease inhibitor cocktail EDTA-free", Roche). Synthesized proteins were subsequently analyzed by SDS-PAGE and autoradiography.
(TIF)

Figure S2 Time course of batch-based (A) and CECF-based (B) expression of eYFP. eYFP was synthesized using an *in vitro* translation system based on insect lysates in the presence of ^{14}C-leucine. Quantification of *de novo* synthesized eYFP was performed by liquid scintillation counting. Protein yields of eYFP are shown in percent with the concentration of the target protein determined after 2 h of incubation set as 100% (batch, + V, - CI). Standard deviations were calculated from triplicate analysis (n = 3).
(TIF)

Figure S3 Time course of ^{14}C-leucine labeled eYFP (supernatant fraction) synthesized in batch and CECF mode. Cell-free reactions using insect lysate were carried out in the presence (+) and absence (−) of insect vesicles (V) and caspase inhibitor (CI). Synthesized proteins were analyzed by SDS-PAGE and autoradiography. Cell-free synthesized eYFP shows a migration pattern corresponding to its expected molecular mass (calculated molecular mass = 29 kDa).
(TIF)

Figure S4 Comparative expression of representative model proteins in *E. coli* and insect cell-free systems. Cell-free synthesis of model proteins was performed using the batch-based *E. coli* cell-free system (EasyXpress Protein Synthesis Kit, Qiagen) and the vesicle-containing insect cell-free system in presence of ^{14}C-leucine. Synthesized proteins were analyzed by SDS-PAGE and autoradiography. Aliquots of the glycoproteins Mel-EPO and Mel-vtPA were subjected to digestion with (+) PNGase F. Black arrows indicate the shift in protein size between glycosylated proteins and proteins after degylcosylation with PNGase F. Only in case of Mel-EPO and Mel-vtPA synthesized in the insect cell-free system, digestion with PNGase F led to a visible reduction of the protein's molecular mass, indicating the successful glycosylation of these proteins. Asterisks are marking the proteins were expression in *E. coli* lysate failed. NTC = No template control; translation reaction without addition of a DNA template.
(TIF)

Figure S5 Glycosylation analysis of cell-free expressed Mel-EPO. Cell-free reactions were carried out in the presence of caspase inhibitor, ^{14}C-leucine and DTT using the insect batch and CECF system (48 h). The autoradiograph shows Mel-EPO in its glycosylated and non-glycosylated form. In addition, a deglycosylation assay was performed using PNGase F.
(TIF)

Figure S6 FRAP analysis of the type-I transmembrane protein Mel-Hb-EGF-eYFP and the fluorescent and soluble protein eYFP. Both proteins were synthesized in standard batch reactions. For confocal microscopy, translation mixtures were excited at 488 nm while fluorescence emission was recorded with a longpass filter in the wavelength range above 505 nm (LSM 510 Meta, Zeiss). Strong emission intensity of *de novo* synthesized Mel-Hb-EGF-eYFP was observed in microsomal structures. In contrast to this observation, fluorescence of eYFP was observed in the cytosolic fraction of the lysate surrounding the vesicles. Samples were analyzed before and after photobleaching. Fluorescent vesicles of Mel-Hb-EGF-eYFP were bleached completely after 500 iterations with 100% laser intensity (argon laser, 488 nm). As expected, no fluorescence recovery was observed after 50 s of incubation, indicating that membrane-embedded Mel-Hb-EGF-eYFP was not delivered by diffusion from the cytosolic surrounding. In contrast, laser exposure of the cytosolic protein eYFP did not result in a detectable photobleaching effect.
(TIF)

Figure S7 Analysis of Mel-vtPA expression and activity. Synthesis of Mel-vtPA was performed in batch mode for 2 h using three different reaction buffers [(1) buffer with DTT; (2) buffer without DTT; (3) buffer without DTT, but supplemented with GSH and GSSG] in combination with insect lysate without DTT, but supplemented with GSH and GSSG. Activity of Mel-vtPA was analyzed using the fibrin-agarose-plate assay. All samples were diluted to 0.5 µg/ml. NTC = No template control; translation reaction without addition of a DNA template. Positive control = Purified full length tPA (Anaspec) (0.5 µg/ml). Standard deviations were calculated from triplicate analysis (n = 3).
(TIF)

Table S1 List of cell-free expressed model proteins.
(DOCX)

Acknowledgments

The authors gratefully acknowledge the contribution of Conny Mascher for cell cultivation and extract preparation. Furthermore, we thank Katja Frede for performing initial experiments on cell-free expression of Mel-vtPA. Plasmids encoding the membrane proteins Hb-EGF and endothelin-B receptor were kindly provided by Prof. Michael Schaefer (Leipzig University, Germany). Moreover, we thank Prof. Edward Darzynkiewicz (Warsaw University) for providing cap analogues. We also wish to express our considerable thanks to Marie Burger for the careful revision of this manuscript.

Author Contributions

Conceived and designed the experiments: MS DAW SK. Performed the experiments: MS RS CS DAW. Analyzed the data: MS RBQ RS CS DAW SK. Contributed reagents/materials/analysis tools: RBQ. Wrote the paper: MS SK.

References

1. Klammt C, Löhr F, Schäfer B, Haase W, Dötsch V, et al. (2004) High level cell-free expression and specific labeling of integral membrane proteins. Eur J Biochem 271: 568–580.
2. Kalmbach R, Chizhov I, Schumacher MC, Friedrich T, Bamberg E, et al. (2007) Functional cell-free synthesis of a seven helix membrane protein: *in situ* insertion of bacteriorhodopsin into liposomes. Journal of Molecular Biology 371: 639–648.
3. Savage DF, Anderson CL, Robles-Colmenares Y, Newby ZE, Stroud RM (2007) Cell-free complements *in vivo* expression of the *E. coli* membrane proteome. Protein Science 16: 966–976.
4. Gourdon P, Alfredsson A, Pedersen A, Malmerberg E, Nyblom M, et al. (2008) Optimized *in vitro* and *in vivo* expression of proteorhodopsin: a seven-transmembrane proton pump. Protein Expression and Purification 58: 103–113.
5. Katzen F, Fletcher JE, Yang J-P, Kang D, Peterson TC, et al. (2008) Insertion of membrane proteins into discoidal membranes using a cell-Free protein expression approach. Journal of Proteome Research 7: 3535–3542.
6. Miot M, Betton J-M (2010) Reconstitution of the Cpx signaling system from cell-free synthesized proteins. New Biotechnology 28: 277–281.
7. Sachse R, Wüstenhagen D, Šamalíková M, Gerrits M, Bier FF, et al. (2013) Synthesis of membrane proteins in eukaryotic cell-free systems. Engineering in Life Sciences 13: 39–48.
8. Rothblatt JA, Meyer DI (1986) Secretion in yeast: Reconstitution of the translocation and glycosylation of α-factor and invertase in a homologous cell-free system. Cell 44: 619–628.
9. Tarui H, Murata M, Tani I, Imanishi S, Nishikawa S, et al. (2001) Establishment and characterization of translation/glycosylation in insect cell (*Spodoptera frugiperda* 21) extract prepared with high pressure treatment. Appl Microbiol Biotechnol 55: 446–453.
10. Suzuki T, Ito M, Ezure T, Shikata M, Ando E, et al. (2006) N-Terminal protein modifications in an insect cell-free protein synthesis system and their identification by mass spectrometry. Proteomics 6: 4486–4495.
11. Ezure T, Suzuki T, Shikata M, Ito M, Ando E, et al. (2007) Expression of proteins containing disulfide bonds in an insect cell-free system and confirmation of their arrangements by MALDI-TOF MS. Proteomics 24: 4424–4434.
12. Suzuki T, Ito M, Ezure T, Shikata M, Ando E, et al. (2007) Protein prenylation in an insect cell-free protein synthesis system and identification of products by mass spectrometry. Proteomics 7: 1942–1950.
13. Kubick S, Gerrits M, Merk H, Stiege W, Erdmann VA (2009) *In vitro* synthesis of posttranslationally modified membrane proteins, In: "Membrane Protein Crystallization" Current Topics in Membranes, Chapter 2 DeLucas L, editor: Elsevier.
14. Shaklee PM, Semrau S, Malkus M, Kubick S, Dogterom M, et al. (2010) Protein incorporation in giant lipid vesicles under physiological conditions. Chembiochem 11: 175–179.
15. Orth JHC, Schorch B, Boundy S, Ffrench-Constant R, Kubick S, et al. (2011) Cell-free synthesis and characterization of a novel cytotoxic pierisin-like protein from the cabbage butterfly *Pieris rapae*. Toxicon 57: 199–207.
16. Avenaud P, Castroviejo M, Claret S, Rosenbaum J, Mégraud F, et al. (2004) Expression and activity of the cytolethal distending toxin of Helicobacter hepaticus. Biochemical and Biophysical Research Communications 318: 739–745.
17. Bechlars S, Wüstenhagen DA, Drägert K, Dieckmann R, Strauch E, et al. (2013) Cell-free synthesis of functional thermostable direct hemolysins of Vibrio parahaemolyticus. Toxicon 76: 132–142.
18. Brödel A, Sonnabend A, Roberts L, Stech M, Wüstenhagen D, et al. (2013) IRES-Mediated Translation of Membrane Proteins and Glycoproteins in Eukaryotic Cell-Free Systems. PLoS One 8: e82234.
19. Brödel AK, Sonnabend A, Kubick S (2013) Cell-free protein expression based on extracts from CHO cells. Biotechnology and Bioengineering 111: 25–36.
20. Madin K, Sawasaki T, Ogasawara T, Endo Y (2000) A highly efficient and robust protein synthesis system prepared from wheat embryos: plants apparently contain a suicide system directed at ribosomes. Proc Natl Acad Sci U S A 97: 559–564.
21. Takai K, Sawasaki T, Endo Y (2010) Practical cell-free protein synthesis system using purified wheat embryos. Nat Protocols 5: 227–238.
22. Vinarov DA, Lytle BL, Peterson FC, Tyler EM, Volkman BF, et al. (2004) Cell-free protein production and labeling protocol for NMR-based structural proteomics. Nat Meth 1: 149–153.
23. Mann M, Jensen ON (2003) Proteomic analysis of post-translational modifications. Nat Biotech 21: 255–261.
24. Kubick S, Schacherl J, Fleischer-Notter H, Royall E, Roberts LO, et al. (2003) *In vitro* translation in an insect-based cell-free system. Swartz JR (Ed) Cell-Free Protein Expression Springer, Berlin Heidelberg New York: 209–217.
25. Stech M, Brödel AK, Quast RB, Sachse R, Kubick S (2013) Cell-Free Systems: Functional Modules for Synthetic and Chemical Biology. Advances in Biochemical Engineering/Biotechnology: Springer Berlin Heidelberg. pp. 67–102.
26. Sachse R, Wüstenhagen D, Šamaliková M, Gerrits M, Bier FF, et al. (2012) Synthesis of membrane proteins in eukaryotic cell-free systems. Engineering in Life Sciences 12: 1–10.

27. Fenz SF, Sachse R, Schmidt T, Kubick S (2014) Cell-free synthesis of membrane proteins: Tailored cell models out of microsomes. Biochim Biophys Acta 1838: 1382–1388.
28. von Groll U, Kubick S, Merk H, Stiege W, Schäfer F (2007) Advances in insect-based cell-free protein expression. In: Kudlicki W, Katzen F, Bennett P, editors. Cell-Free Protein Expression Landes Bioscience.
29. Brödel AK, Raymond JA, Duman JG, Bier FF, Kubick S (2013) Functional evaluation of candidate ice structuring proteins using cell-free expression systems. Journal of Biotechnology 163: 301–310.
30. Merk H, Gless C, Maertens B, Gerrits M, Stiege W (2012) Cell-free synthesis of functional and endotoxin-free antibody Fab fragments by translocation into microsomes. BioTechniques 53: 153–160.
31. Stech M, Merk H, Schenk J, Stöcklein W, Wüstenhagen D, et al. (2012) Production of functional antibody fragments in a vesicle-based eukaryotic cell-free translation system. J Biotechnol 164: 220–231.
32. Kim D-M, Swartz JR (1999) Prolonging cell-free protein synthesis with a novel ATP regeneration system. Biotechnology and Bioengineering 66: 180–188.
33. Spirin A, Baranov V, Ryabova L, Ovodov S, Alakhov Y (1988) A continuous cell-free translation system capable of producing polypeptides in high yield. Science 242: 1162–1164.
34. Baranov VI, Morozov I, Ortlepp SA, Spirin AS (1989) Gene expression in a system on the preparative scale. Gene 84: 463–466.
35. Kim D, Choi C (1996) A semicontinuous prokaryotic coupled transcription/translation system using a dialysis membrane. Biotechnol Prog 12: 645–649.
36. Katzen F, Chang G, Kudlicki W (2005) The past, present and future of cell-free protein synthesis. Trends Biotechnol 23: 150–156.
37. Spirin AS (2004) High-throughput cell-free systems for synthesis of functionally active proteins. Trends in Biotechnology 22: 538–545.
38. Oh I-S, Kim D-M, Kim T-W, Park C-G, Choi C-Y (2006) Providing an oxidizing environment for the cell-free expression of disulfide-containing proteins by exhausting the reducing activity of *Escherichia coli* S30 extract. Biotechnology Progress 22: 1225–1228.
39. Bulleid NJ, Bassel-Duby RS, Freedman RB, Sambrook JF, Gething MJ (1992) Cell-free synthesis of enzymically active tissue-type plasminogen activator. Protein folding determines the extent of N-linked glycosylation. Biochem J 286: 275–280.
40. Gething MJ, Adler B, Boose JA, Gerard RD, Madison EL, et al. (1988) Variants of human tissue-type plasminogen activator that lack specific structural domains of the heavy chain. EMBO J 7: 2731–2740.
41. Van Noorden CJF (2001) The history of Z-VAD-FMK, a tool for understanding the significance of caspase inhibition. Acta Histochemica 103: 241–251.
42. Granelli-Piperno A, Reich E (1978) A study of proteases and protease-inhibitor complexes in biological fluids. J Exp Med 148: 223–234.
43. Ali M, Suzuki H, Fukuba T, Jiang X, Nakano H, et al. (2005) Improvements in the cell-free production of functional antibodies using cell extract from protease-deficient Escherichia coli mutant. J Biosci Bioeng 99: 181–186.
44. Lazebnik Y, Kaufmann S, Desnoyers S, Poirier G, Earnshaw W (1994) Cleavage of poly(ADP-ribose) polymerase by a proteinase with properties like ICE. Nature 371: 346–347.
45. Sixt BS, Hiess B, König L, Horn M (2012) Lack of Effective Anti-Apoptotic Activities Restricts Growth of Parachlamydiaceae in Insect Cells PLOSOne 7: e29565.
46. Tseng Y-K, Wu M-S, Hou RF (2008) Induction of apoptosis in SF21 cell line by conditioned medium of the entomopathogenic fungus, Nomuraea rileyi, through Sf-caspase-1 signaling pathway. Archives of Insect Biochemistry and Physiology 68: 206–214.
47. Qin M, Zhang J, Wang W (2006) Effects of Disulfide Bonds on Folding Behavior and Mechanism of the beta-Sheet Protein Tendamistat. Biophysical Journal 90: 272–286.
48. Darby NJ, Morin PE, Talbo G, Creighton TE (1995) Refolding of bovine pancreatic trypsin inhibitor via non-native disulphide intermediates. Journal of Molecular Biology 249: 463–477.
49. Rietsch A, Beckwith J (1998) The genetics of disulfide bond metabolism. Annual Review of Genetics 32: 163–184.
50. Anfinsen CB (1973) Principles that Govern the Folding of Protein Chains. Science 181: 223–230.
51. Collen D, Lijnen HR (2009) The Tissue-Type Plasminogen Activator Story. Arterioscler Thromb Vasc Biol 29: 1151–1155.
52. Kierzek AM, Zaim J, Zielenkiewicz P (2001) The Effect of Transcription and Translation Initiation Frequencies on the Stochastic Fluctuations in Prokaryotic Gene Expression. Journal of Biological Chemistry 276: 8165–8172.
53. Sonenberg N, Hinnebusch A (2009) Regulation of Translation Initiation in Eukaryotes: Mechanisms and Biological Targets. Cell 136: 731–745.
54. Bushell M, Poncet D, Marissen WE, Flotow H, Lloyd RE, et al. (2000) Cleavage of polypeptide chain initiation factor eIF4GI during apoptosis in lymphoma cells: characterisation of an internal fragment generated by caspase-3-mediated cleavage. Cell Death Differ 7: 628–636.
55. Marissen WE, Lloyd RE (1998) Eukaryotic translation initiation factor 4G is targeted for proteolytic cleavage by caspase 3 during inhibition of translation in apoptotic cells. Mol Cell Biol 18: 7565–7574.
56. Gradi A, Svitkin YV, Imataka H, Sonenberg N (1998) Proteolysis of human eukaryotic translation initiation factor eIF4GII, but not eIF4GI, coincides with the shutoff of host protein synthesis after poliovirus infection. Proc Natl Acad Sci USA 95: 11089–11094.
57. Bushell M, Wood W, Clemens MJ, Morley SJ (2000) Changes in integrity and association of eukaryotic protein synthesis initiation factors during apoptosis. Eur J Biochem 267: 1083–1091.
58. Satoh S, Hijikata M, Handa H, Shimotohno K (1999) Caspase-mediated cleavage of eukaryotic translation initiation factor subunit 2alpha. Biochem J 342: 65–70.
59. Marissen WE, Guo Y, Thomas AAM, Matts RL, Lloydi RE (1999) Identification of Caspase 3-mediated Cleavage and Functional Alteration of Eukaryotic Initiation Factor 2α in Apoptosis. J Biol Chem 275: 9314–9323.
60. Oh I-S, Lee J-C, Lee M-s, Chung J-h, Kim D-M (2010) Cell-free production of functional antibody fragments. Bioprocess and Biosystems Engineering 33: 127–132
61. Jiang X, Ookubo Y, Fujii I, Nakano H, Yamane T (2002) Expression of Fab fragment of catalytic antibody 6D9 in an *Escherichia coli in vitro* coupled transcription/translation system. FEBS Letters 514: 290–294.
62. Kawasaki T, Gouda MD, Sawasaki T, Takai K, Endo Y (2003) Efficient synthesis of a disulfide-containing protein through a batch cell-free system from wheat germ. European Journal of Biochemistry 270: 4780–4786.
63. Park C-G, Kim T-W, Oh I-S, Song JK, Kim D-M (2009) Expression of functional Candida antarctica lipase B in a cell-free protein synthesis system derived from Escherichia coli. Biotechnol Prog 25: 589–593.
64. Rouf SA, Moo-Young M, Chisti Y (1996) Tissue-type plasminogen activator: Characteristics, applications and production technology. Biotechnology Advances 14: 239–266.
65. Lin A, Kimura R, Miller W (1993) Production of tPA in recombinant CHO cells under oxygen-limited conditions. Biotechnol Bioeng 42: 339–350.
66. Qiu J, Swartz J, Georgiou G (1998) Expression of active human tissue-type plasminogen activator in *Escherichia coli*. Appl Environ Microbiol 64: 4891–4896.
67. Kim DY, Chaudhry MA, Kennard ML, Jardon MA, Braasch K, et al. (2013) Fed-batch CHO cell t-PA production and feed glutamine replacement to reduce ammonia production. Biotechnol Prog 29: 165–175.
68. Yin G, Swartz JR (2004) Enhancing multiple disulfide bonded protein folding in a cell-free system. Biotechnology and Bioengineering 86: 188–195.
69. Chen RH, Huang C Jr, Newton BS, Ritter G, Old LJ, et al. (2009) Factors affecting endotoxin removal from recombinant therapeutic proteins by anion exchange chromatography. Protein Expression and Purification 64: 76–81.
70. Ramya R, Mohana Subramanian B, Sivakumar V, Senthilkumar R, Sambasiva Rao K, et al. (2011) Expression and Solubilization of Insect Cell-Based Rabies Virus Glycoprotein and Assessment of Its Immunogenicity and Protective Efficacy in Mice. Clin Vaccine Immunol 18.
71. Naftzger C, Takechi Y, Kohda H, Hara I, Vijayasaradhi S, et al. (1996) Immune response to a differentiation antigen induced by altered antigen: A study of tumor rejection and autoimmunity. Proceedings of the National Academy of Sciences 93: 14809–14814.
72. Haberstock S, Roos C, Hoevels Y, Dötsch V, Schnapp G, et al. (2012) A systematic approach to increase the efficiency of membrane protein production in cell-free expression systems. Protein Expr Purif 82: 308–316.
73. Schwarz D, Klammt C, Koglin A, Löhr F, Schneider B, et al. (2007) Preparative scale cell-free expression systems: new tools for the large scale preparation of integral membrane proteins for functional and structural studies. Methods 41: 355–369.
